# Turbulent flow interacting with flexible trawl net structure including simulation catch in flume tank

**DOI:** 10.1038/s41598-023-33230-y

**Published:** 2023-04-17

**Authors:** Bruno Thierry Nyatchouba Nsangue, Hao Tang, Wei Liu, Liuxiong Xu, Fuxiang Hu

**Affiliations:** 1 College of Marine Sciences, Shanghai Ocean University, 999 Huchenghuan Road, Lingang New District, Shanghai, 201306 People’s Republic of China; 2 National Engineering Research Center for Oceanic Fisheries, Shanghai, 201306 People’s Republic of China; 3 Key Laboratory of Oceanic Fisheries Exploration, Ministry of Agriculture and Rural Affairs, Shanghai, 201306 People’s Republic of China; 4 The Key Laboratory of Sustainable Exploitation of Oceanic Fisheries Resources, Ministry of Education, Shanghai Ocean University, Shanghai, 201306 People’s Republic of China; 5 Center for Polar Research, Shanghai Ocean University, Shanghai, 201306 People’s Republic of China; 6 Department of Marine Biosciences, Tokyo University of Marine Science and Technology, Minato, Tokyo, 108-8477 Japan

**Keywords:** Environmental sciences, Ocean sciences, Engineering

## Abstract

The interaction between fluid and the midwater trawl with stocked catches is extremely complex, but essential to improve the understanding of the drag force acting on the trawl, the behavior of the fishing structure during a trawling process, and to predict its selectivity process. The present study assesses the turbulent characteristics inside and around the midwater trawls with catch and without catch linked to its fluttering motion. The analysis is based on three-dimensional electromagnetic current velocity meter measurements performed in the multiple points inside and outside different parts of a 1/35 scaled midwater trawl model with the aim of access the main turbulent flow structure inside and around the gear. Time-averaged normalized flow velocity fields and turbulent flow parameters were analyzed from the measured flow data. Furthermore, Fourier analysis was conducted by watching the time–frequency Power spectrum content of instantaneous flow velocities fields, the fluttering trawl motions, turbulent kinetic energy, and momentum flux. Based on successive analyzes of mean flow characteristics and turbulent flow parameters, it has been demonstrated that the presence of catch inside the trawl net impacts the evolution of unsteady turbulent flow by creating large trawl fluttering motions that strongly affect the flow passage. The results showed that the time-averaged normalized streamwise and transverse flow velocities inside and around the trawl net with catch were 12.41% lower compared with that obtained inside and around the trawl without catch. The turbulent length scale and turbulent Reynolds number obtained in the different part of the trawl net with catch were about 33.05% greater than those obtained on the trawl net without catch, confirming that the unsteady turbulent flow developing inside and around the midwater trawl is influence by the catch and liner. It is observed that the motions of both the trawl without catch and the trawl with catch are mainly of a low-frequency activity and another component related to unsteady turbulent flow street. A complex fluid–structure interaction is then demonstrated where the fluttering motions of the trawl net affect the fluid flow inside and around trawl net, the fluid force, turbulent pattern, and simultaneously, the periodic unsteady turbulent flow influence the trawl motions.

## Introduction

Nowadays, the midwater trawl fisheries face several constraints such as the continuous increase in fuel price and decrease in fish stocks^1,4^. To solve these problems, the optimum structural design of the trawl is necessary to reduce the hydrodynamic force, reduce the by-catch, improve the juvenile escape rate, and improve the trawl efficiency^4–6^. In this case, the researchers and fishing gear designers achieved the reduction of hydrodynamic force by the reduction in twine diameter, increase in mesh size or the use of square meshes, replacement of twine material, and the modification of the shape of the trawl net^7–9^. For the improvement of trawl selectivity, they predominantly examined the mesh size in relation to the fish size^10,11^. However, to better understand the engineering performance and the selectivity of trawl nets, the coupled dynamics of the unsteady turbulent flow developing inside and around the trawl net and fluttering motions of trawl should be considered as the main factors affecting trawl performance^12–14^. Indeed, the intense motion of the water can severely deform the trawl net leading to the development of unsteady turbulent flows^15,16^. These turbulent flows are caused by the presence of a liner and catch, which limit the flow through the trawl net^10,11,17–19^. This development of the unsteady turbulent flow will generate strong vertical drag pressure on the trawl and create unstable motions of the trawl net^13^.

The analysis of the complex interaction between unsteady turbulent flow and the moving trawl structure including catch is a fundamental scientific topic that has drawn increased attention and studies in scientific research in both simulation and experimental work^20–22^. This analysis provides a better understanding of the flow behavior and oscillatory motions of the trawl net, thus affords relevant information for the analysis of hydrodynamic forces and selectivity via the fish response, such as the herding response or escape behavior^10,14^. Indeed, the fluid flow loads inside and around a trawl net structure can be clarified in static and dynamic loads, and are directly related to its elastic and dynamic instabilities^13,23^. The former is due to the vertical dynamic pressure variation of the flow fields along with the trawl structure, and the latter is associated with pressure and velocity fluctuations due to the vortex shedding, turbulent boundary layer, and the turbulent flow in the trawl wake. Due to the constraints imposed by these unsteady turbulent flows, the presence of the catch inside the trawl codend and the waterbody pressure on the trawl net, the dynamic horizontal and vertical motions of the trawl have been developed^24–26^. Furthermore, the provenances of such oscillatory motions for an immersed trawl net in an unsteady turbulent flow are due firstly, to the generated vibrations caused by local hydrodynamic effects such as fluctuating velocities and the turbulence in flow as we mentioned above^13^. Secondly due to environmental changes and some factors such as current flow, fishing vessel motion by wave or wind, natural underwater flows, and an adjustment of the warp length causing a deviation of the trawl position from the depth of the krill schools^10^. Finally, they are due to the generated vibrations caused by the variation in warp tension and the motions of the deformable trawl structure itself that can strongly modify its shape. These trawl oscillations can cause relative motions of the outer and inner of the trawl, leading to a higher hydrodynamic force on the trawl system and a lower size selectivity^3,4,27^.

Recently, much progress has been made in the analysis of the complex interaction between a flexible fluttering trawl net, turbulent flow, and catches via numerical simulations, flume tank experiments, and sea trials. Ziembo^28^ proposed equations that can be used on the flow boundary conditions of a trawl, describing the transition from a laminar to a turbulent flow due to the low Reynolds number in a specified area of the flow around the trawl wall contours. Paschen^29^ investigated the effect of a fluid blockage on the flow field through a pelagic trawl net and found that fluid–structure interaction between this structure and turbulent flow can strongly affect the selectivity of fishing gear. Kim^10,30^ analysed the turbulent flow inside the trawl codend during the sea trial by performing one-point measurements using the 3D Acoustic Doppler Velocimeter (ADV) and demonstrating that the presence of the turbulent flow inside the trawl codend affected the fish behavior. Druault et al.^20,24^ and Bouhoubeiny et al.^25^ used time-resolved particle image velocimetry (TRPIV) combined with a motion tracking technology for the measurement of the instantaneous motion of the mean flow field from unsteady turbulent flow developing around random oscillated structure deformable fishing net and bottom trawl to investigate the turbulent boundary layer flow. They demonstrated the feasibility of the Proper Orthogonal Decomposition (POD) procedure to extract the instantaneous mean flow field and confirmed that the transverse and longitudinal oscillations of the fishing net and trawl structure induce opening variability which may have a significant effect on the escape of fish and the engineering performance of the trawl net. Druault and Germain^13^ characterized the flow in the unsteady wake developed behind the fluttering codend structure and found that the vortex shedding that occurs in the wake zone around the codend can cause trawl oscillations. Tang et al.^31^ have developed a numerical model to analyze the flow field through the trawl by proposing the deformation of the trawl. They propose a numerical model simulation to calculate a drag force on the trawl net. Chen and Yao^32^ also adopted the hybrid volume method to describe fluid trawl interaction. First, they modelled the trawl net and the flow field based on the lumped mass method and finite volume method, separately. Then, they adopted a hybrid volume method (HVM) to model the fluid–structure interaction between the net and surrounding water. Thierry et al.^6,23^ analyzed the behavior of the turbulent flow developing inside and around the bottom trawl based on the numerical simulation and flume tank experiment. They found that the trawl motions linked to this turbulent flow considerably modified the opening and the geometrical shape of the mesh component on the trawl net, which then strongly influenced the spatial and temporal fluctuations of the selected performance parameters of the trawl net.

Despite the existing work on the subject and the available data, there is no relevant conclusion in the experimental studies evaluating the effect of the catch on the fluid–structure interaction of a midwater trawl structure. Thus, the present study is motivated by the need to characterize the unsteady turbulent flow nature inside and around the midwater trawl, to clarify the influence of the catch and trawl motions on the engineering performance and to understand the catch process and fish selectivity in the real conditions. Further motivation is provided by the current lack of detailed quantitative information on the interaction between the oscillatory motions of the midwater trawl with catch and the hydrodynamic behavior of the turbulent flow inside and around it.

The focus of this study is to analyze the hydrodynamics and associated trawl motions and to provide significant information that could be extrapolated to a full-scale midwater trawl in some conditions. More precisely, this study characterizes the flow inside and around different parts of the trawl net and analyses the phenomenon of the fluttering trawl motions. We then quantify the effect of catch on the turbulent flow via turbulent kinetic energy, higher-order moments, and further analyse the power spectrum content of the velocity field, turbulent kinetic energy, and Reynolds stress. A series of experiments were conducted to measure the flow velocity field inside and around the 1:35 scale of Antarctic krill trawl models using the electromagnetic current velocity meter (ECVM) approach. The findings are expected to contribute to the improvement of midwater trawl performance and trawl selectivity control.

## Methods and materials

### Description of the 1:35 scaled midwater trawl model and experiment setup

**Figure 1:**
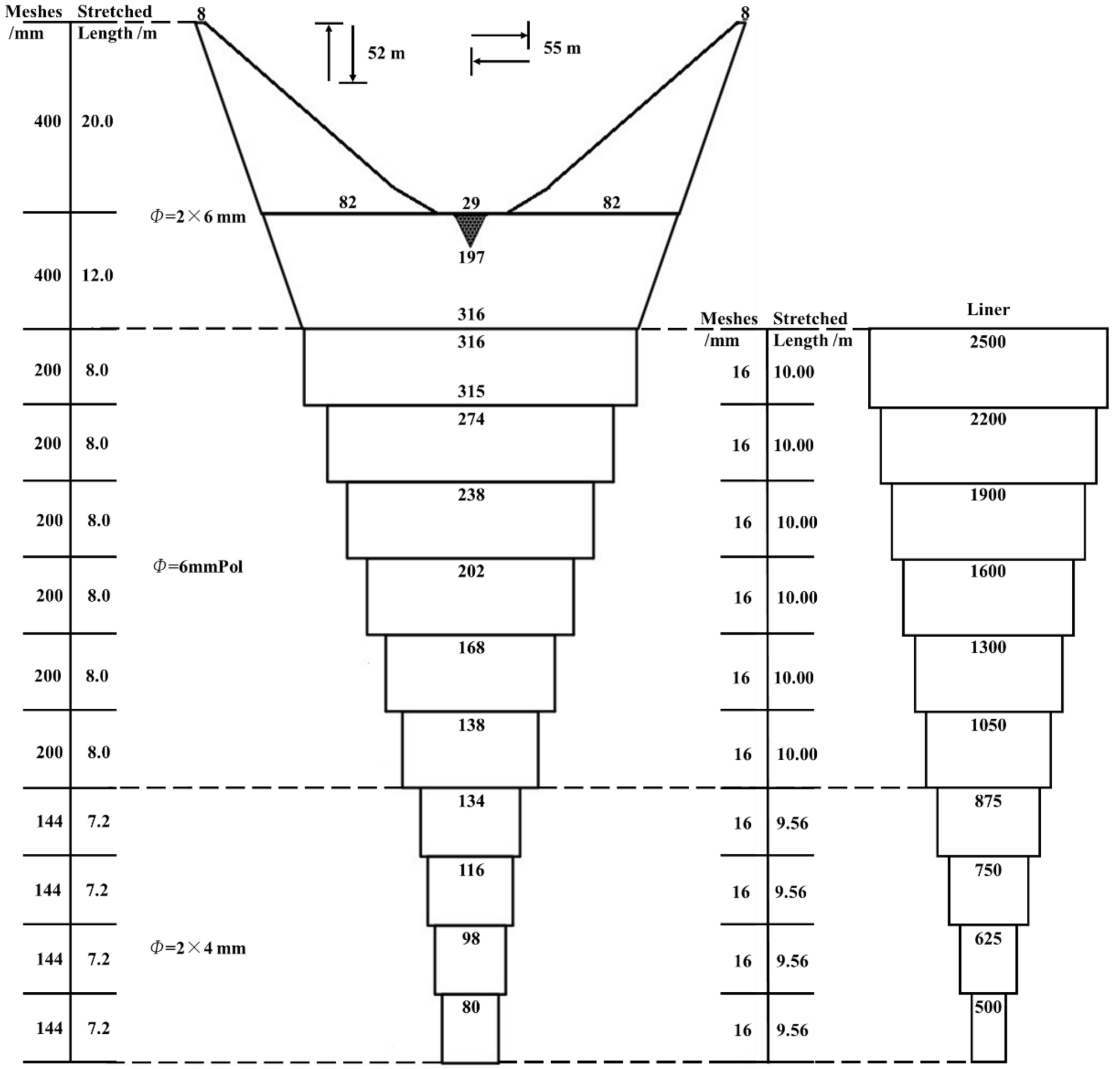
Schematic net plan of Antarctic krill trawl in full-scale.

A 1:35 scaled model of a four-panel midwater trawl that is commonly used in the Antarctic fisheries by Chinese fishing vessels “Long Teng” of China National Fisheries Corporation was selected as the trawl design for this study (Fig. 1). Overall, the circumference of the trawl net at the mouth was 8.848 m, the trawl length (L) was 3.85 m, the headline length was 1.59 m, the bridle length was 1.8 m, and the fishing line length was 1.568 m, consisting of chains with the weight of 21 g. The trawl model was built using polyethylene (PE) twine materials with diamond-shaped mesh. The twine diameter of this model was from 0.8 to 1.2 mm varying in mesh size from 80 mm in the trawl wing and the first section of the trawl body to 40 mm in the remaining trawl body sections and the codend (Fig. 1). This trawl model was combined with a liner constructed using polyamide (PA) material, with a mesh of 10 mm and a twine diameter of 0.2 mm. To ensure the vertical opening of the trawl model, floats with a total buoyancy of 1.5 N were placed on the headline and two heavy bobs of 48.98 g each were attached under the wing-end. The horizontal opening was ensured by two vertical bar considered as the trawl doors in which the two bridles were connected.

The design of the model was based on the modified Tauti’s law developed by Hu et al.^33^. Thus, the scale model was designed in such a way as to approach the geometric, kinematic, dynamic, turbulent, and force law of a full-scale trawl (More detail can be seen in Thierry et al.^34^ and Tang et al.^35^). In this study, the length scale λ was assumed to be 1/35 and the mesh size or twine diameter scale $\lambda ^{\prime}$ was 1/5. Therefore, the turbulent flow velocity (*u*) and the drag force (*Fd*) of the full scale can be estimated using the following equations:

1 $$\frac{{u_{M} }}{{u_{F} }} = \left( {\lambda ^{{\prime}^{n} \lambda }} \right)^{{\frac{1}{2 - n}}}$$

2 $$Fd_{F} = \frac{{Fd_{M} }}{{\lambda ^{\prime}W\lambda^{2} }}$$

where F and M are the full-scale and model, respectively, *n* represents the exponent of the function *Cd* = *k* ${Re}^{-n}$ referring to the study of Hu et al.^33^ and assumed to be 0.15 for the midwater trawl, and W= $\frac{{\rho }_{sM-{\rho }_{M}}}{{\rho }_{sF}-{\rho }_{F}}$ with ${\rho }_{s}$ and *ρ* represent the material density and water density, respectively.

**Figure 2:**
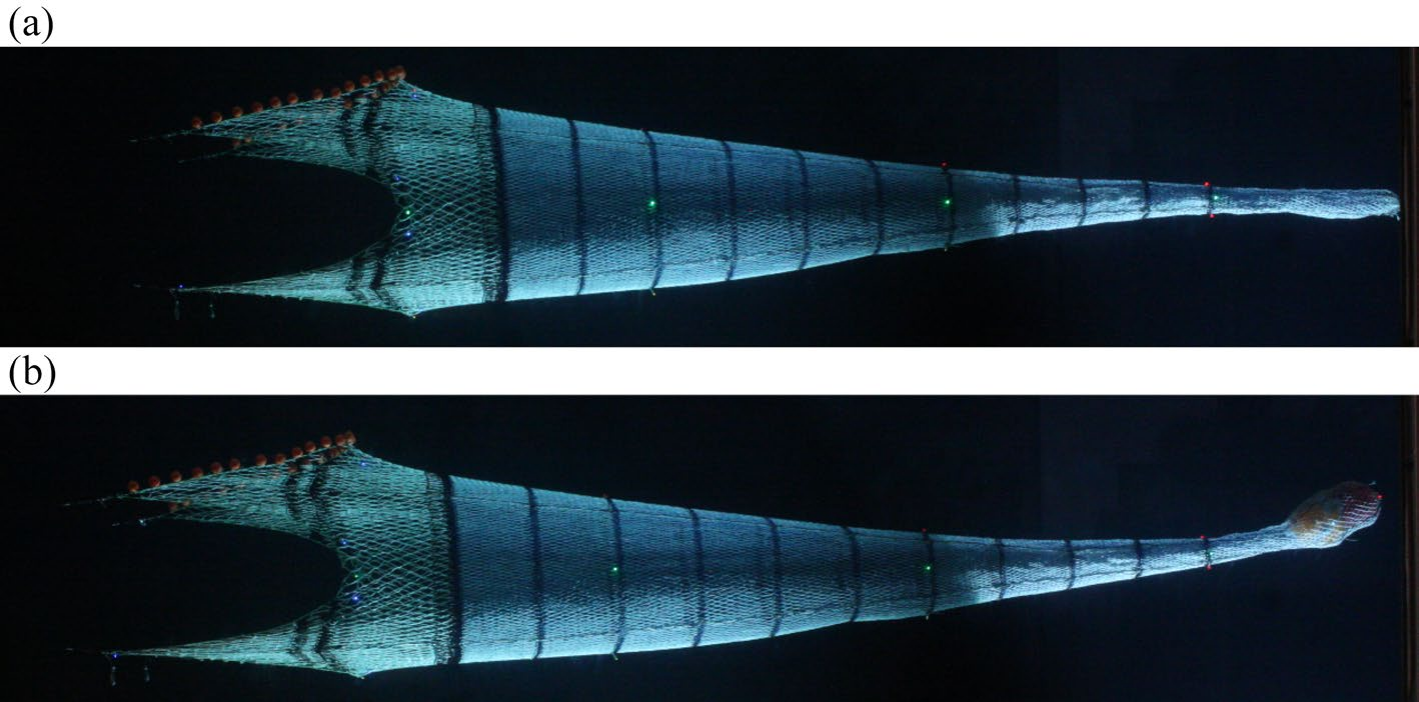
Trawl model with liner in the flume tank of TUMSAT: (**a**) model without catch and (**b**) model with catch.

During the measurement, the fish catch was simulated using small plastic balls filled with an average of 0.045 kg of water each, a total of 350 filled balls represented a total catch of 15.75 kg (Fig. 2).

The experiments were performed at the Tokyo University of Marine Sciences and Technology (TUMSAT) circulation flume tank. The flume tank working section was 9.0 m long, 2.2 m wide and 1.6 m depth, containing ~ 150 tons of freshwater. Four contra-rotating impellers with a diameter of 1.6 m and using constant-speed hydraulic delivery pumps were utilized to drive the flow, the above delivered a flow speed that could be varied from 0.1 to 2.0 m/s, an acceleration flow that could be varied from 1.0 to 5.0 cm/${\mathrm{s}}^{2}$, and an amplitude oscillation of the flow velocity of $\pm$ 0.5 m/s^36^. The measurements were performed at a fixed streamwise velocity of 0.36 m/s, a door spread of 1.8 m, and with a turbulence level of 5%. This turbulence level corresponded to the one computed in a flume tank without fishing gear where the flow was uniform^23,36^. Using Reynolds decomposition, the turbulence level has been determined and it is of an order of 4.63%. During the flow measurement, the turbulent boundary layer developed around the side wall of the flume tank did not affect the measurement of the flow field on the different points inside and around the trawl net as confirmed by Thierry et al.^23^ for the bottom trawl or Jonsson et al.^37^ on the analysis of the hydrodynamic characteristics on 12 different benthic biological flumes. The flow meter (propeller tachometer) was placed at 1.2 m directly in front of the trawl mouth to detect the current flow velocity. At the fixed streamwise velocities, the mean net mouth height (*H*) of the trawl net measured was 0.79 m. The water density of the flume tank was 999.8 kg/m^3^, and the water temperature was maintained in the range of 17.6–18.4 ºC.

In this study, the trawl codend shape was assumed to be a sphere, and the diameter of this sphere (*d*) was measured during the experiments and the reported values were 0.11 m and 0.32 m for the trawls without catch and with catch, respectively. Thus, to evaluate the effect of structure and catch on the turbulent, the Reynolds number was established using the twine diameter and diameter of the sphere^11,13^:

3 $$R_{e } = \frac{{ u_{0 } L}}{\nu },$$

where ${u}_{0}$ is the flow velocity, L is the length in m, ν is the kinematic viscosity, and ${R}_{e}$ is the Reynolds number. The Reynolds numbers used during experimental flow measurement are described in Table 1.

**Table 1:** Corresponding Reynolds number.

Model nets	$R_{e}$ as function of twine diameter	$R_{e}$ as function of trawl codend diameter
Trawl without catch	4071.6	59,208.3
Trawl with catch	4071.6	172,196

### Flow measurement system

**Figure 3:**
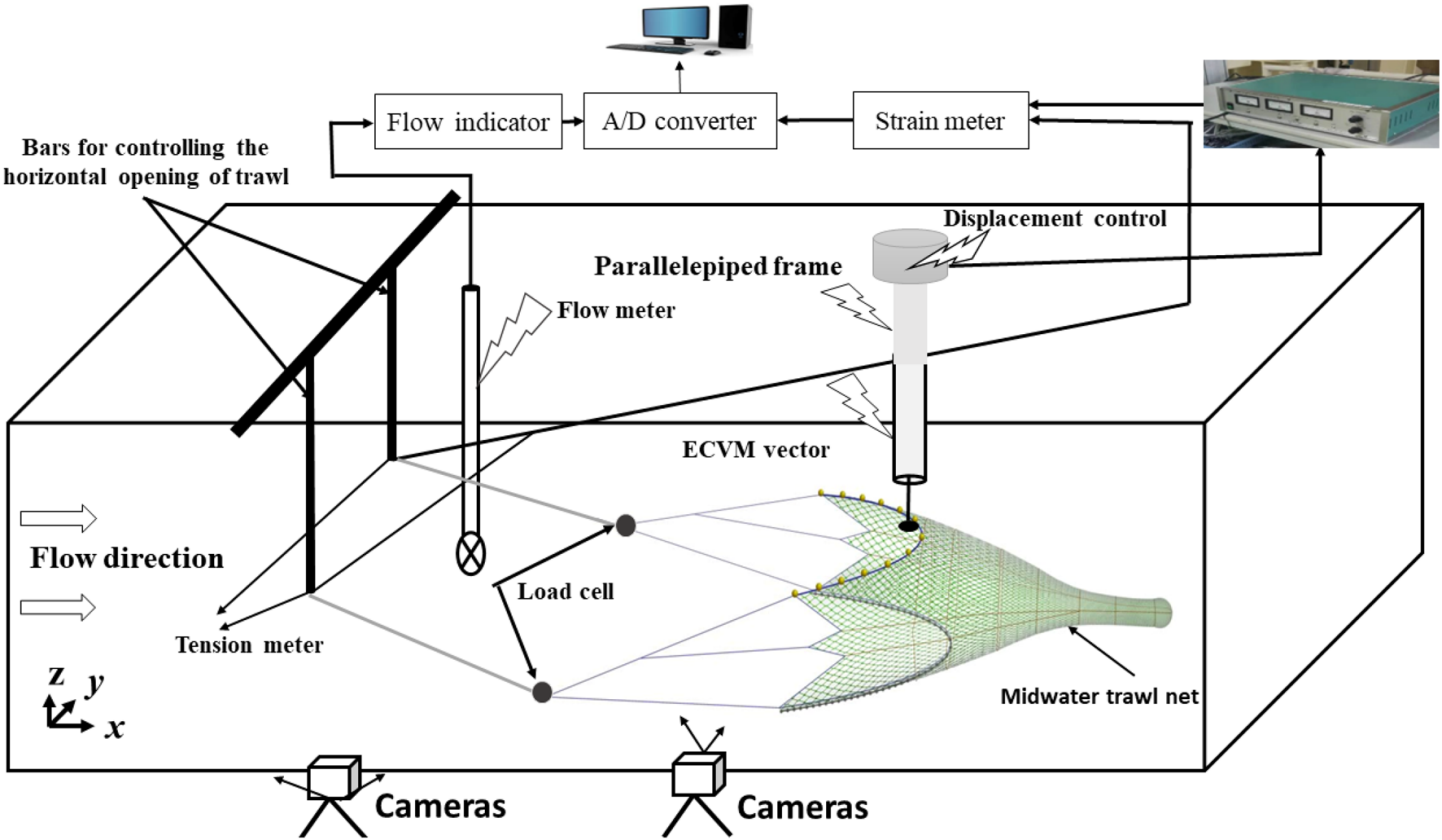
Experimental process of the scale model of the krill trawl tested in the flume tank.

**Figure 4:**
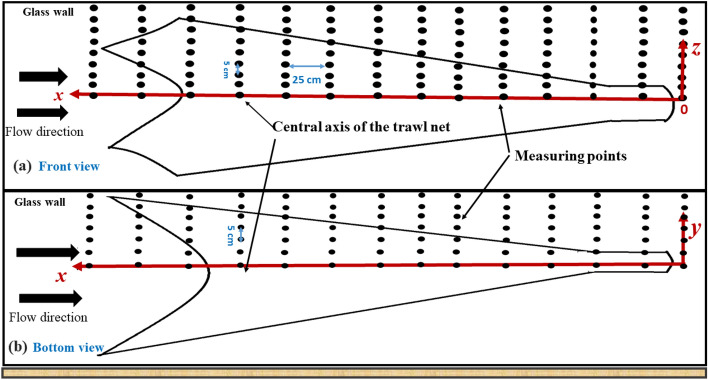
ECVM measurement point locations as shown in black circles in (**a**) Front view and (**b**) Bottom view.

To characterize the flow inside and around the different points of the midwater trawl, three-component ECVM ACM3-RS techniques were used. The ECVM devise used was manufactured by JFE Advantech Co., Ltd, Nishinimiya, Japan. This is an electromagnetic induction type with a diameter of 34 mm and a length of 420 mm. The detecting parts were characterized by a diameter of 6 mm, a length of 18 mm, a specified accuracy of 0.5 cm/s (2%), a resolution of 0.1 cm/s, and a zero-point stability of ± 0.15 cm/s (according to specifications provided by the manufacturer). Instantaneous velocity fields were measured in the symmetrical plane with respect to the centerline of the trawl that divided the trawl net into two equal parts (Fig. 4). The origin was placed at the end point of the trawl codend on its central axis (Fig. 4), the direction of the flow was aligned with the *x-*axis, and the direction perpendicular to the water surface was aligned with the *z*-axis and y-axis (Figs. 3 and 4). The measurement of the flow field inside and around the different parts of the trawl started at a measurement point of 0 cm (behind the trawl codend) on the centerline on the trawl net representing the *x*-axis. The middle of door spread (0.9 m) was the *y*-axis and the transverse direction(*z*-axis) on the central line of the trawl net (about 0.25 m) (Figs. 3 and 4). With this configuration, flow velocity inside and around the gear was defined as the velocity relative to the top and side panels of the trawl net. This velocity has three components: longitudinal direction (*u*), perpendicular direction (*v*), and vertical direction (*w*). In our study, the values of the *v* component were neglected because they were very low. In the study, the *x*-direction and *z*-direction are called streamwise and transverse directions, respectively. The ECVM device measured instantaneous velocity field in the three axes and the two cameras placed on the front and bottom views of the flume allowed the measurement of the coordinates of the different points of the trawl in three directions. Fourteen points were equidistantly between the point behind of the trawl codend tail and the point in front of the wing-end, each distance along the *x*-axis was 25 cm. Meanwhile, along the *y*-axis, seven points were set on one side of the water path in the middle of the door spread, within 5 cm from the central axis. Along the *z*-axis, nine points were set within 5 cm of the central axis. The same measurements were done on the other symmetrical side of the trawl net on the *y*-axis(-y) and *z*-axis(-z) and the velocity measurements were almost the same as those obtained on the positive axes. Due to the low mesh size of the liner, the mesh on the different measurement points was increased by combining the four meshes on the liner, each size of 40 mm. The ECVM probe was attached to the parallelepiped steel frame that allowed it to asses different parts of the trawl net, keep it in the orthogonal position, and protect the device sensor from obstacles. The combined ECVM probe and steel frame were mounted on an instrumentation rack that could be moved in three dimensions (i.e., flow direction, perpendicular direction, and depth direction) at 5 cm steps, the vector device was held in an orthogonal position, and the device sensor was protected from obstacles^23^. At each measurement point, 500 data points of velocity were recorded for a duration of 125 s at a sampling rate of 4 Hz; thus, the three velocity components were assessed every 125 s. These measurements were run three times to assess the repeatability of the measurement. According to the Reynolds decomposition, each instantaneous velocity component is separated into a mean value and fluctuating part:

4 $$u\left( {x, \, z, \, t} \right) = \overline{{u\left( {x,z} \right)}} + u^{\prime} \, \left( {x, \, z, \, t} \right)$$

where $\overline{{u\left( {x,z} \right)}}$ is the time average flow velocity defined by* :*

5 $$\overline{{u\left( {x,z} \right)}} = \frac{1}{T}\mathop \smallint \limits_{0}^{T} u\left( {x, z, t} \right)dt$$

where [*0; T*] is the averaging period, and (*u', w’) represents* its associated fluctuating part.

Due to the random motion of the trawl structure and the unsteady nature of flow developing inside and around the trawl net, the Reynolds stress tensor denoted $\tau_{ij}$ are used to analyse the momentum flux. It is defined as follows:

Reynolds normal stress components:

6 $$\tau_{xx} = \overline{{u^{{\prime}{2}} }} ,$$

7 $$\tau_{yy} = \overline{{w^{{\prime}{2}} }} ,$$

Reynolds shear stress component

8 $$\tau_{xy} = \overline{u^{\prime}w^{\prime}} ,$$

### Description of the measurement of the trawl motions

As we mentioned above, a series of videos describing the trawl fluttering motions were taken from the front and bottom view of the flume tank observation port as indicated in Fig. 3. A three-minute recording was made of the trawl net during each test case with the two video cameras kept in a fixed position and set to constant zoom and focus settings. The system contains two cameras placed in the front and at the bottom of the flume tank windows, which enables it to track the motion of objects in the large volume of the flume tank. The markers were placed at different parts of the trawl to follow the motions of the whole trawl. The video cameras used to record the videos had a frequency of 4 Hz per frame image and a resolution of 1920 × 1080 ${\mathrm{pixels}}^{2}$(manufactured by Dantec Hi-sense, with a focal lens length of 60 mm). In order to obtain the fluttering motion of the two trawls, we used the first minute (60 s) of the three-minute recording. Thus, to obtain the trawl motions, a series of images (240) were firstly captured at 0.25 s each from the recorded video footage. These images were imported in MATLAB R2019B software to extract the coordinates of the characteristic points on each trawl net part based on a plane-coordinates. To ensure the data reliability, a standard bar was used in different locations to calibrate the measurements and to mitigate the effect caused by strains in the camera’s lens, water refraction, and parallax (more detail can be seen in^23^). In this study, only the temporal evolution of the codend tail motions was determined in order to analyse the effect of catch on the trawl motions.

### Description of the higher-order moments

The higher-order moment developed in this study provides additional information regarding the nature of instantaneous flow structures developing inside and around midwater trawl net.

#### Turbulent flux and the gradient of turbulent kinetic energy flux

The turbulent flux uses an approximate expression diffusive transport inside this trawl net. This turbulent flux is used as a function of the turbulent kinetic energy, dissipation, gradient of the turbulent stresses for the turbulent modelling inside and around the midwater trawl, and the effect of catch on this turbulent structure. The gradient of turbulent kinetic energy flux indicates the nature probability density distribution of the turbulent fluctuation and exhibits the rate of the variation of the kinetic energy owing to the diffusion^38,39^. Finally, the gradient of turbulent kinetic energy flux allows observing the motion of the turbulent structure and its effect on the trawl performance.

The turbulent flux of the shear stress through the trawl net in streamwise and transverse directions are defined as:

9 $$D_{u} = \frac{{\overline{{ u^{\prime}w^{{\prime}{2}} }} }}{{u_{0}^{3} }},\quad D_{w} = \frac{{\overline{{ w^{\prime}u^{{\prime}{2}} }} }}{{u_{0}^{3} }}.$$

The turbulent kinetic energy flux is used to statistically analyse the properties of the unsteady turbulent flow inside and around the whole trawl net. They can be defined as follows:

10 $$f_{ku} = 0.5(\overline{{u^{\prime}u^{\prime}u^{\prime}}} + \overline{u^{\prime}w^{\prime}w^{\prime})} /u_{0}^{3}$$

11 $$f_{kw} = 0.5(\overline{{w^{\prime}w^{\prime}w^{\prime}}} + \overline{u^{\prime}u^{\prime}w^{\prime})} /u_{0}^{3}$$

#### Turbulent kinetic energy, turbulent length scale, and Taylor-scale Reynolds number

The turbulent kinetic energy (TKE) allows us to evaluate the way the turbulent structure is spatially distributed after its generation through the trawl net and the different mechanisms involved. It can be written as follows:

12 $$k = \frac{1}{2}\left( {\overline{{u^{{\prime}{2}} }} + \overline{{w^{{\prime}{2}} }} } \right)$$

In this study, the turbulent length scale and the Taylor-scale Reynolds number was used to characterize the unsteady turbulent flow and to compare it between different flows developing inside and around the different part of the two midwater trawl nets. Thus, the turbulent length scale and Taylor-scale Reynolds number are determined as follows^39–42^:

13 $${\uplambda }_{{\text{T}}} = \sqrt {15\nu \sigma_{u}^{2} /\varepsilon } ,$$

where ${\sigma }_{u}$ is the root mean square (RMS) of streamwise turbulent fluctuation and can be estimated as follows:

14 $$\sigma_{u} = \sqrt {\frac{{\mathop \sum \nolimits_{i = 1}^{n} u^{{\prime}{2}} }}{n}}$$

$\varepsilon$ is the energy dissipation rate and it is given by the following equation^43^:

15 $$\varepsilon = 15\nu \overline{{\left( {\frac{\partial u^{\prime}}{{\partial x}}} \right)^{2} }}$$

From $k = \frac{1}{2}\left( {\overline{{u^{{\prime}{2}} }} + \overline{{w^{{\prime}{2}} }} } \right)$ and by assuming that we analyze the flow behavior in the *x*-direction, we can derive by $k = \frac{2}{2}\left( {\overline{{u^{{\prime}{2}} }} } \right)$ and;

16 $$\uplambda _{{\text{T}}} \approx \sqrt {15\nu k/\varepsilon }$$

Taylor-scale Reynolds number can be determined based on the length scale $\uplambda _{{\text{T}}}$ and the corresponding velocity scale as follows:

17 $$R_{{\uplambda }} = \frac{{u^{\prime}\uplambda _{{\text{T}}} }}{\nu }$$

### Fast Fourier transform

In this study, the fast Fourier transform (FFT) was used to evaluate the frequency spectrum of the signal of turbulent flow parameters (flow velocity, turbulent energy, and Reynolds stress), trawl motions, and converted the time domain to the frequency domain of each time series parameters of N samples (240 for the case of trawl motion in each direction and 500 for each turbulent parameters). This method was based on Welch’s method using a window and was implemented using MATLAB R2019B software. The use of this method for the implementation of the FFT is because the interaction between the trawl structure and the unsteady turbulent flow allows the need for the much longer signals in order to evaluate the predominant frequency peaks with sufficient precision. In this case, the Fourier analysis is dependent on a periodic function which may be expressed as the sum of waveforms of different frequencies^43,44^. Indeed, the FFT converts waveform data in the time domain into the frequency domain. FFT accomplishes this by breaking down the original time-based waveform into a series of sinusoidal terms, each with a unique magnitude, frequency, and phase. This process, in effect, converts a waveform in the time domain that is difficult to describe mathematically, into a more manageable series of sinusoidal functions that when added together, reproduce the original waveform exactly. Plotting the amplitude of each sinusoidal term versus its frequency creates a power spectrum, which is the response of the original waveform in the frequency domain^45^. A waveform data such as the turbulent velocity and trawl motions can be expressed as:

18 $$x\left( t \right) = \mathop {x\left( t \right) = \sum }\limits_{n = 0}^{N - 1} x_{n} e^{ - i2\pi nt/T}$$

The frequency of such a function can be defined by the discrete Fourier transform (DFT):

19 $$X_{wDFT} \left( k \right) = \mathop \sum \limits_{n = 0}^{N - 1} x\left( n \right)w_{n}^{{\left( {n - 1} \right)\left( {k - 1} \right)}}$$

where ${w}_{n}$= ${e}^{-i2\pi t/N}$, $x(n)$ is the signal, *i* is the imaginary unit, and *N* is the number of samples.

However, for the sine signal, the magnitude spectrum can be defined by $\left|{X}_{wDFT}(k)\right|$ in which the Fourier transform can be seen as a maximum likelihood estimation for the amplitude. In this case, the signal amplification is estimated by multiplying the normalized frequency with $\frac{2}{N}$.

Thus, the power spectrum that represents the mean-square amplitude of the different signals such as turbulent parameters, and trawl motions can be estimated using the Fourier transform of each measurement. This power spectrum is determined by splitting the measurement into overlapping sequences using the method called Welch’s method and defined as^46^:

20 $$\hat{P}_{w} \left( {e^{jw} } \right) = \frac{1}{KLU}\mathop \sum \limits_{i = 0}^{k - 1} \left| {\mathop \sum \limits_{n = 0}^{i - 1} w\left( n \right)x\left( {n + iD} \right)e^{ - jnw} } \right|^{2}$$

where ${\widehat{P}}_{w}({e}^{jw})$ is the estimated power spectrum. Each sequence of length L is overlapping D points with the successive sequence. For *N* data points, *K* is the number of sequences needed to cover all data points.* U* = $\frac{1}{N}{\sum }_{n=0}^{N-1}{\left|w(n)\right|}^{2}$ and $w(n)$ represent the data window applied to modify each frequency. In this study, the sampling frequency was assumed to be 4 Hz.

### Ethical approval

The authors have read, contributed to preparing the manuscript and attest to the validity and legitimacy of the data and its interpretation. This manuscript does not contain any studies with human participants or animals performed by any of the authors.

## Results

### Mean flow characterization inside and around the midwater trawl net

**Figure 5:**
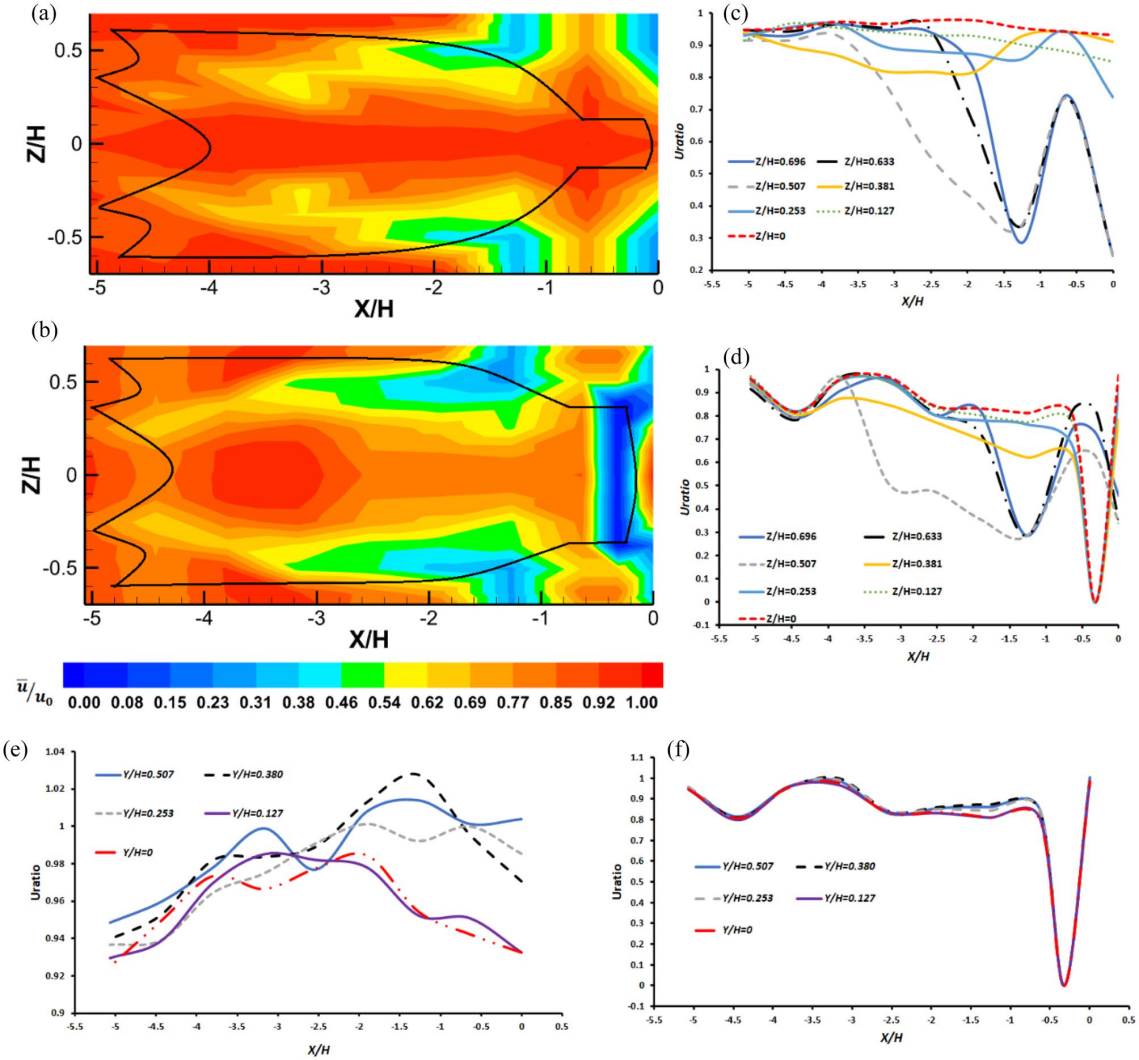
Mean streamwise flow velocity ($\overline{u}/u_{0}$) develops inside and around midwater trawl net (**a**) Trawl without catch and (**b**) Trawl with catch, and the velocity curves along the *X/H* direction as function of Z*/H* at Y*/H* = 0 (**c** and **d**) and *Y/H* at *Z/H* = 0 (**e** and **f**) for trawls without catch and with catch, respectively.

**Figure 6:**
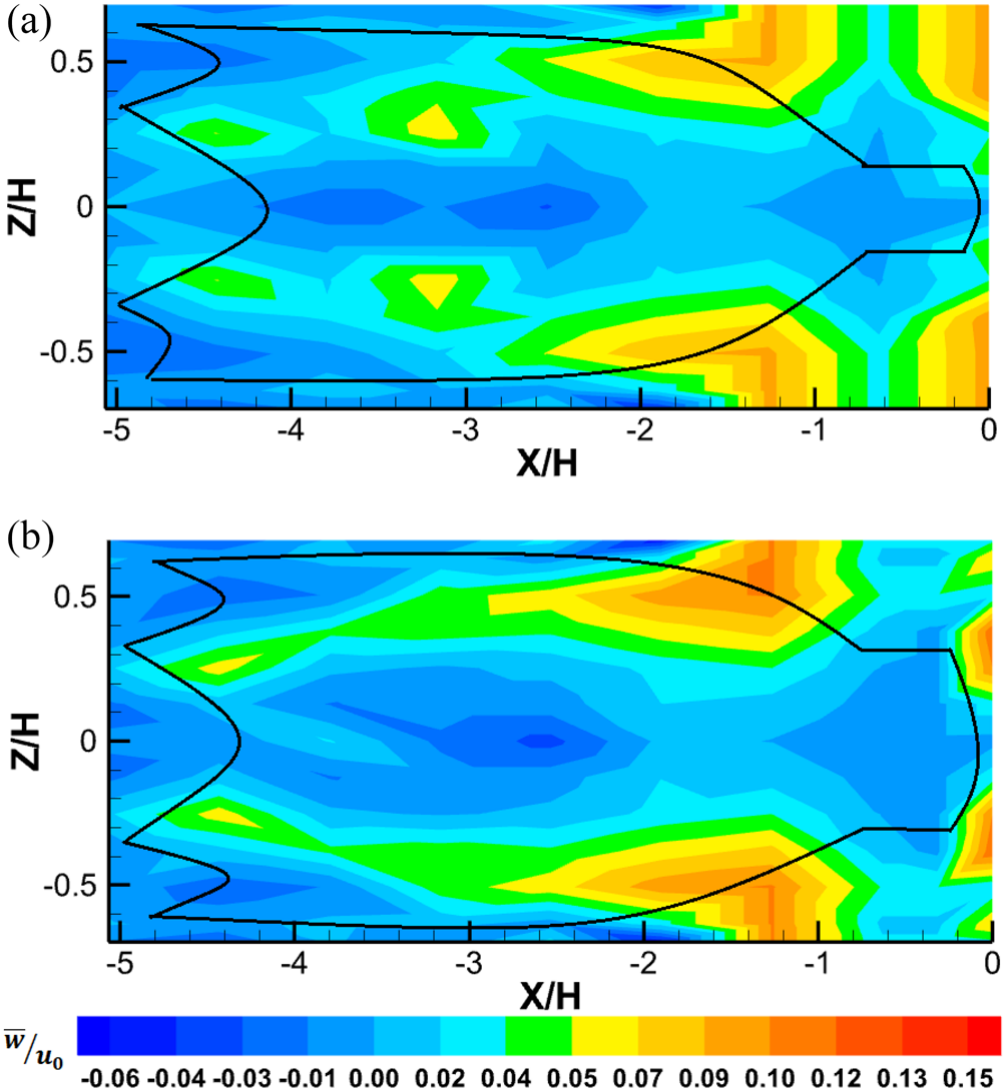
Mean transverse flow velocity ($\overline{w}/u_{0}$) develops inside and around midwater trawl net (**a**) Trawl without catch and (**b**) Trawl with catch at the front view.

Figures 5 and 6 shows the time-averaged normalized streamwise (*u*) and transverse (*w*) velocity inside and around the midwater trawl net present in front view. In this figure, the black line indicates the positions of the trawl net structure. Inside and around the trawl wing (− 5.06 $<$ *X/H* $<$ − 3.79), the trawl body (− 3.79 $<$ *X/H* $<$ − 1.27), and the codend (− 1.27 $<$ *X/H* $<$ 0), the streamwise flow velocity field recovered the input streamwise velocity (${u}_{0}$) by about 0.156–0.964 ${u}_{0}$, 0.156–0.989 ${u}_{0}$, and 0.262– 0.951 ${u}_{0}$, respectively, for the trawl without catch (Fig. 5a,c,e). For the trawl with catch, the distribution of the streamwise velocity ratio varied from 0.155–0.986 ${u}_{0}$, 0.156–0.971 ${u}_{0}$, and 0–0.908 ${u}_{0}$ inside and around the trawl wing, the trawl body, and the codend, respectively (Fig. 5b,d,f). Observation showed that the flow passage changed as a function of the catch and the oscillatory motions of the midwater trawl. At *Z/H* = 0.127 and 0, the flow velocity curve decreased slightly with variations of 2.6–3.06% and velocity reductions ranged from 2.21 to 15.01% along the *X/H* direction inside the trawl without catch (Fig. 5c). However, for the trawl with catch, the flow measurements made on the inside points of the trawl wing and the first part of the trawl body (− 5.06 < *X/H* < − 2.53) had a velocity ratio between 0.793 and 0.968 and a velocity deficit varying from 3.18 to 20.70% (Fig. 5d). The streamwise velocity ratio was ~ 0.809–0.831 with a deficit drops between 16.88 and 19.91% in the trawl net with catch, near the codend (− 1.89 < *X/H* < − 0.31) on the centerline. Therefore, for *Z/H* > 0.253 from *X/H* = − 3.79 inside the trawl net, the streamwise flow velocity tends to greatly decrease along an increasing *X/H* until it reaches a minimum flow velocity near the codend structure and results in a velocity reduction ranging from 5.3 to 33.3% (Fig. 5d).

At *X/H*–*Y/H* axis, the flow velocity curves of the trawl without catch increase at *Z/H* > 0.127 and $\overline{u}/u_{0}$ vary from 0.94 to 1.02. On the same axis at *Z/H* = *0.127* and 0*, *$\overline{u}/u_{0}$ increases and reaches maximum values of *0.98* at *X/H* = − *3.37 (Y/H* = *0.127)* and *X/H* = − *1.89(Y/H* = *0.127)* then, decreases to the minimum value of 0.93 (see Fig. 5e)*.* For the trawl net with catch, the values of $\overline{u}/u_{0}$ are very close at all *Y/H* points, resulting in a gap of less than 2% (Fig. 5f).

Figures 5 and 6 also show time-averaged normalized streamwise (*u*) and transverse (*w*) values inside and around the trawl with catch are 12.41% lower, compared with that obtained inside and around the trawl without catch (see Figs. 5a,b and 6). The streamwise flow velocities are lower outside the trawl body from the seventh section and the codend, compare to those obtained inside and around the other parts of the trawl net (Fig. 5). While the transverse flow velocities are greater around the codend and the trawl body from the seventh section, compare to those obtained inside and around the trawl wing and the other trawl body sections (Fig. 6). On average, the values of *w* were 95.91% and 95.17% lower compare to the values of *u* inside and around the trawls without catch and with catch, respectively.

The analysis of the time-averaged normalized streamwise (*u*) indicates that the turbulent flow that develops inside and around the midwater trawl net is probably turbulent boundary layer flow. More precisely, these develop around the trawl body and the codend on the *X/H–Z/H* axis, this is represented by different layers (i.e., different colors) stating from low flow velocity near the structure, to high flow velocity away from the structure (Fig. 5). The flow fields inside and around the trawl wing and trawl body also correspond to the turbulent flow due to the trawl wake being more observed inside the trawl body both for the two trawls. These turbulent flows are important inside and outside the last half of the trawl body and the codend due to lower flow velocities around these sections, and more specifically, around the trawl with catch (Fig. 5b).

**Figure 7:**
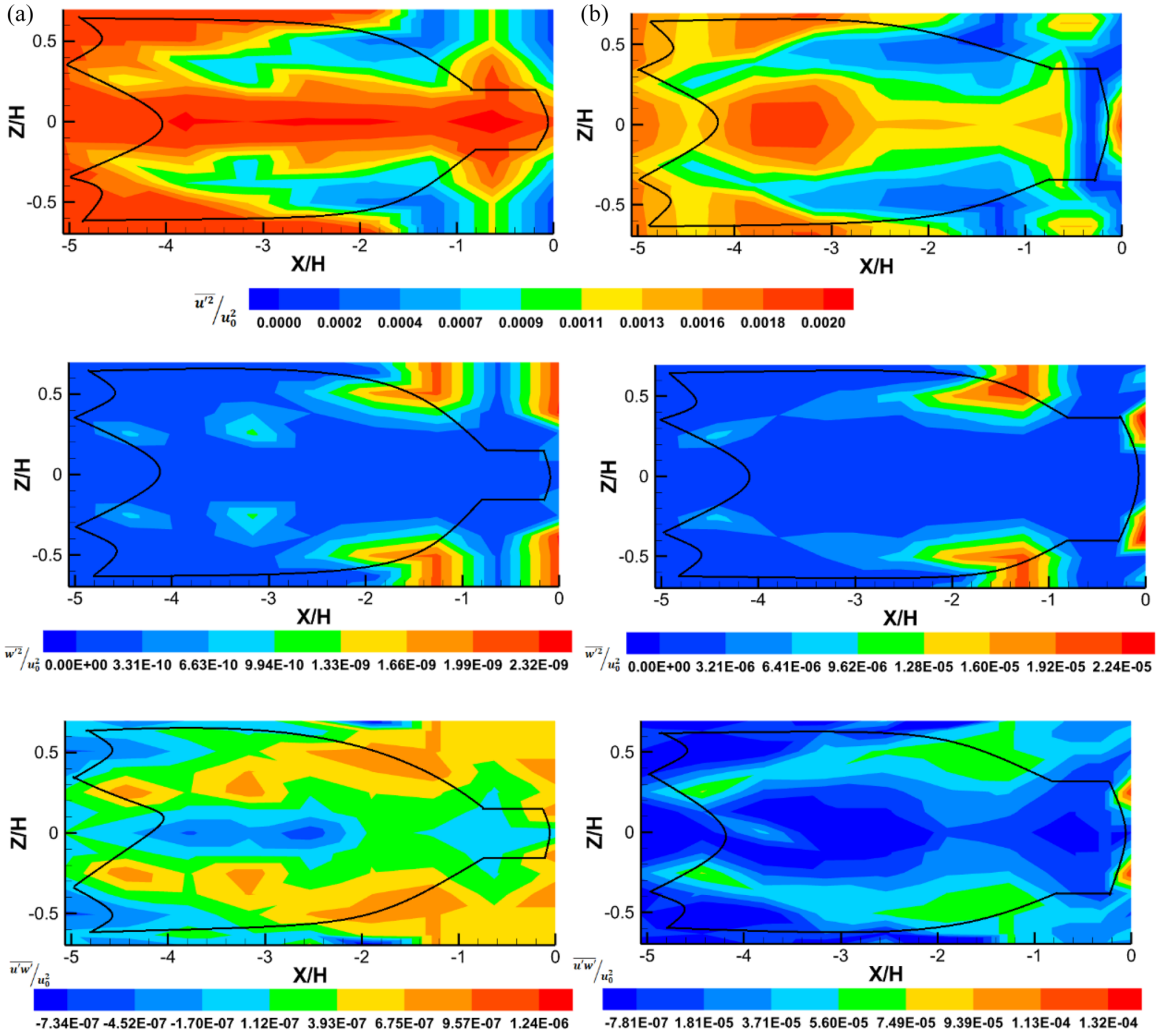
Reynolds stresses tensor components of the flow field inside and around (**a**) trawl net without catch and (**b**) trawl net without catch: (up) ($\overline{{u^{{\prime}{2}} }} /u_{0}^{2}$), (middle) $\overline{{w^{{\prime}{2}} }} /u_{0}^{2}$ , and (bottom) $\overline{{u^{\prime}w^{\prime}}} /u_{0}^{2}$.

Figure 7 compares the momentum flux (Reynolds stresses) between the two trawl nets. The results indicated that the Reynolds stresses are symmetric at the structure centreline (*Z/H* = 0) and that the average normal components are greater than the average shear components for both the trawls without catch and with catch (Fig. 7a,b). The normal Reynolds stress $\overline{{u^{{\prime}{2}} }} /u_{0}^{2}$ values in the internal section of the trawl are 97.92% higher compared to around and near the trawl structure both for the trawls without catch and with catch (Fig.7a,b). However, lower distribution was observed inside and around the trawl body near the structure at − 3.79 < *X/H* < − 1.89 and around the codend at − 0.63 < *X/H* < 0 for both the trawl without catch and with catch. This lower distribution was also observed inside the codend of the trawl due to the presence of catches (Fig. 7b). Note that the lower values of $\overline{{u^{{\prime}{2}} }} /u_{0}^{2}$ correlate to a high-velocity gradient and large turbulence production due to instabilities shear and turbulent boundary layers develops through the two trawl nets (Figs. 5, 7a,b). The mean $\overline{{u^{{\prime}{2}} }} /u_{0}^{2}$ inside and around trawl without catch is 16.45% greater than that obtained through trawl with catch (Fig. 7a,b).

The values of $\overline{{w^{{\prime}{2}} }} /u_{0}^{2}$ are higher around the trawl body last section on the lower and upper side (− 2.09 < *X/H* < − 1.12), and around the codend (− 0.25 < *X/H* < 0) for both the trawls net without catch and with catch (Fig. 7a,b). These results also show that the value of $\overline{{w^{{\prime}{2}} }} /u_{0}^{2}$ obtained inside and around the trawl with catch are 99.98% greater than those obtained inside and around the trawl without catch (Fig. 7a,b).

For the trawl without catch, the$\overline{{u^{\prime}w^{\prime}}} /u_{0}^{2}$ magnitudes are positively distributed inside the shear layer on the trawl wake observed inside and around all the parts of the trawl net except inside the trawl wing and the first section of the trawl body (− 4.43 < *X/H* < − 2.53) (See the bottom of Fig. 7a). For the trawl with catch, negative distributions existed inside the trawl wing and the first part of the trawl body at − 5.06 < *X/H* < − 2.53, and inside the codend at − 1.4312 < *X/H* < 0 (See the bottom of Fig. 7b). This indicates lower level of turbulent mixing and is therefore a negligible momentum exchange in the flow through these parts of the midwater trawl net. The magnitude $\overline{{u^{\prime}w^{\prime}}} /u_{0}^{2}$ is 98.69% higher for the trawl with catch compared to that observed inside and around the trawl without catch. Furthermore, the dissimilarity in the distribution of $\overline{{u^{{\prime}{2}} }} /u_{0}^{2}$, $\overline{{w^{{\prime}{2}} }} /u_{0}^{2}$, and $\overline{{u^{\prime}w^{\prime}}} /u_{0}^{2}$ for different trawl nets, indicated that the occurrence of different turbulent transport mechanisms (Fig. 7). These mechanisms involved flow passage through the trawl net undergoing systematic accelerations or decelerations and appear to be associated with the dynamics of turbulent structures developed inside and around the trawl net with catch. The systematic acceleration or decelerations lead to an increase in Reynolds stress due to the greater trawl motions observed behind the codend of the trawl with catch (Fig. 7b). In addition, it was found that the relationship between the normal Reynolds stress components was $\sqrt {\overline{{u^{{\prime}{2}} }} /u_{0}^{2} } > \sqrt {\overline{{w^{^{\prime}2} /u_{0}^{2} }} }$ and that $\overline{{u^{\prime}w^{\prime}}} /u_{0}^{2}$ $\ne$ 0, which confirms the turbulent character of the flow through the midwater trawl structure.

### Analysis of the trawl fluttering motions

**Figure 8:**
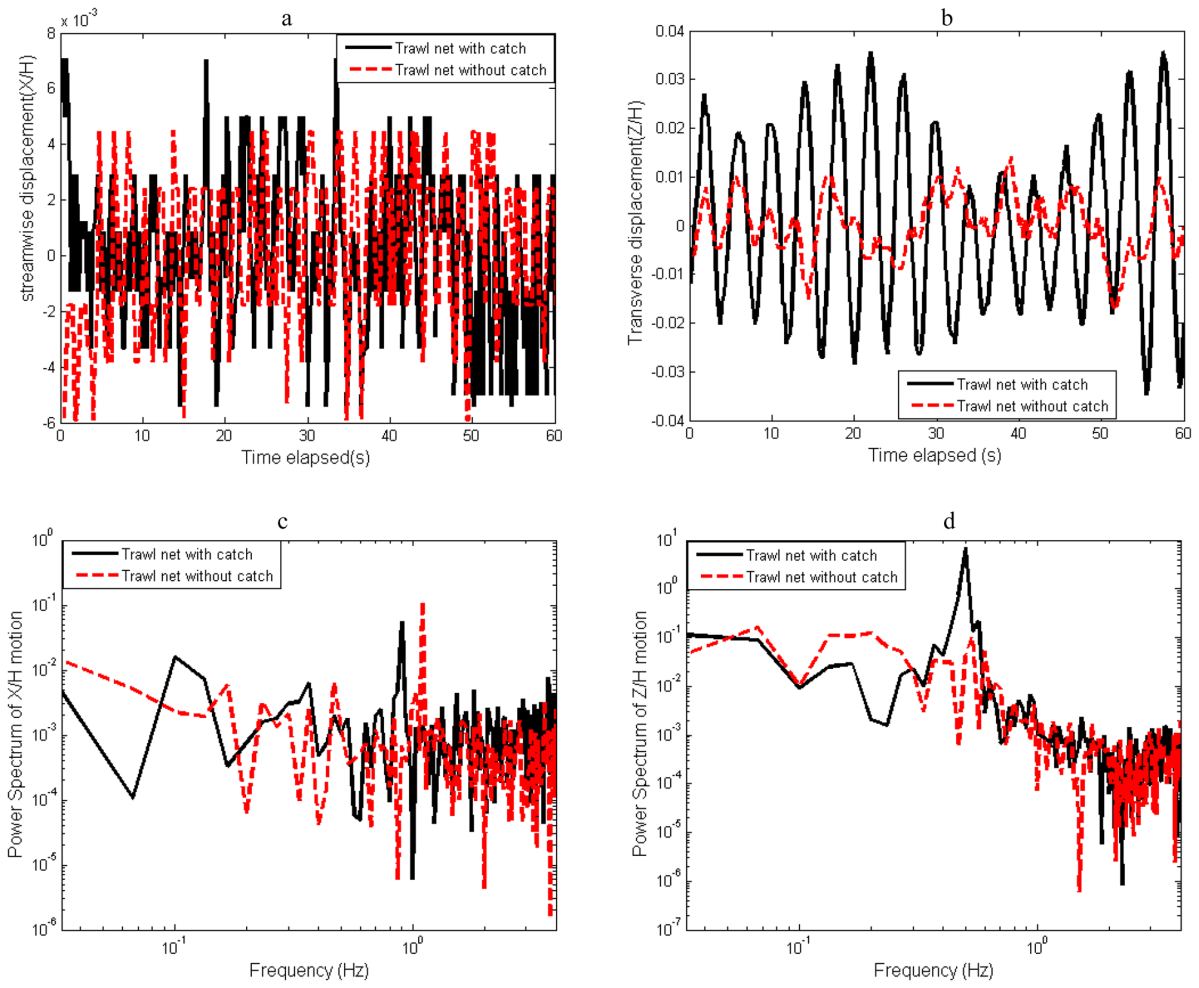
(**a**) Fluctuations of the x-structure motion normalized with the net mouth height (H). (**b**) Fluctuations of the normalized z-structure motion. (**c**) Spectral representation of x-structure motion, and (**d**) Spectral representation of z-structure motion (in a log–log scale).

The trawl motion in the two directions is influenced by the catch size and is characterized by quasi-periodic oscillations (Fig. 8a,b). The structure of the transverse oscillation amplitude for the trawl with catch is more than six times greater than the one related to the streamwise oscillations. While, for the trawl without catch, the structure’s *z*-oscillation amplitude is two times higher than the *x*-oscillation. For the trawl with catch, peak to peak vibration of streamwise and transverse oscillation are 0.0063*H* and 0.032* H*, respectively, which correspond to ± 1.67% and ± 5.37% of *H.* While, for the trawl without catch, the peak-to-peak vibrations of the oscillations are 0.0032*H* and 0.013* H*, respectively, for the streamwise and transverse motions, corresponding to ± 1.43% and ± 2.93% of *H* (Fig. 8a,b)*.* The maximum amplitudes of the transverse and streamwise oscillations for the trawl with catch were greater than those of the trawl without catch (Fig. 8a,b)*.*

**Figure 9:**
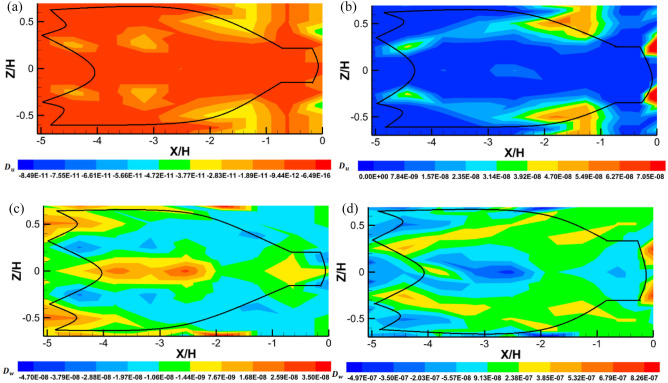
Turbulent flux components of the flow field inside and around midwater trawl net Trawl without catch (**a** and **c**) and Trawl with catch (**b** and **d**).

In Fig. 8c,d, the spectral content of the structure’s oscillations is given in a log–log scale obtained both for the motion of trawls without catch and with catch. The highest frequency peak of the streamwise oscillations obtained at low-frequency components of ${f}_{1}$ = 0.033 and 0.10 Hz for the trawls without catch and with catch, respectively. The second frequency peak on the x-oscillations is at ${f}_{2}$ = 1.10 and 0.90 Hz for the trawl without catch and with catch, respectively (Fig. 8c). For the transverse structure’s oscillations, the highest frequency peaks for the trawls without catch and with catch were at low frequencies of ${f}_{1}$ = 0.066 and 0.033 Hz, respectively (Fig. 8c). On the transverse structure’s oscillations, the second frequency peaks were at ${f}_{2}$ = 0.53 and 0.50 Hz for the trawls without catch and with catch, respectively (Fig. 8). In this case, the interaction between ${f}_{1}$ and ${f}_{2}$ induces an additional frequency ${f}_{3}$= ${f}_{2 }-{f}_{1 }=$ 1.067 and 0.80 Hz for the trawls without catch and with catch, respectively, on *x*-oscillations. On *z*-oscillations, ${f}_{3}$ was 0.370 and 0.467 Hz for the trawls without catch and with catch, respectively. The spectra obtained in the trawl with catch exhibited a dominant low-frequency component compared to that of the trawl without catch. When regarding the power spectrum content, the amplitude of the transverse oscillation’s spectra was 29.65% and 74.11% higher than that of streamwise oscillation spectra for the trawls without catch and with catch, respectively (Fig. 9c,d).

### Analysis of the higher-order moments

#### Analysis of the turbulent flux and gradient of turbulent kinetic energy flux

Figure 9a,b shows the turbulent flux** (**${D}_{u}$) inside and around the midwater trawl both without catch and with catch. For the trawl without catch, the distribution of ${D}_{u}$ are uniformly higher with negative values varying from – 3.6×${10}^{-11}$ to − 6.06×${10}^{-16}$, the lower distributed values of − 5.6×${10}^{-11}$ to − 3.6×${10}^{-11}$ obtained inside and around the end-part of the codend are exempted due to the free motions on the *X/H–Z/H* axis. For the trawl with catch, ${D}_{u}$ distribution varies consistently, with the lower values varying from − 4.18×${10}^{-3}$ to 1.13×${10}^{-7}$ except the values varying from 1.52×${10}^{-7}$ to 2.31 × ${10}^{-7}$, inside the trawl wing at − 5.06 < *X/H* < − 4.43 (Fig. 9b). The magnitude value of ${D}_{u}$ was higher inside and around the trawl net with catch compared to that inside and around the trawl without catch (Fig. 9). This means that the diffuse transport of the Reynolds stress, the energy dissipation, and the gradient turbulent stress developing inside and around the trawl net were strongly affected by the disturbance of the flow passage through the trawl.

Figures 9c and d show the turbulent flux** (**${D}_{w}$) in transverse direction inside and around the midwater trawl both with catch and without catch. The values inside and around the trawl without catch, ${D}_{w}$ consist of the negative values varying from − 3.88×${10}^{-8}$ to − 1.43 × ${10}^{-9}$. However, the maximum and positive values of 7.78×${10}^{-9}$ to 3.55×${10}^{-8}$ were inside the trawl body at − 4.43 < *X/H* < − 2.26, and inside the end-part of the codend due to an unsteady turbulent flow inside this trawl net (Fig. 9c). For the trawl with catch, the ${D}_{w}$ were distributed evenly with lower values varying from − 4.46×${10}^{-7}$ to 2.38×${10}^{-7}$ inside and around all the parts of the trawl with catch with exception to the end part of the codend (6.79×${10}^{-7}$ to 8.26 × ${10}^{-7}$) (Fig. 9d)*.*

**Figure 10:**
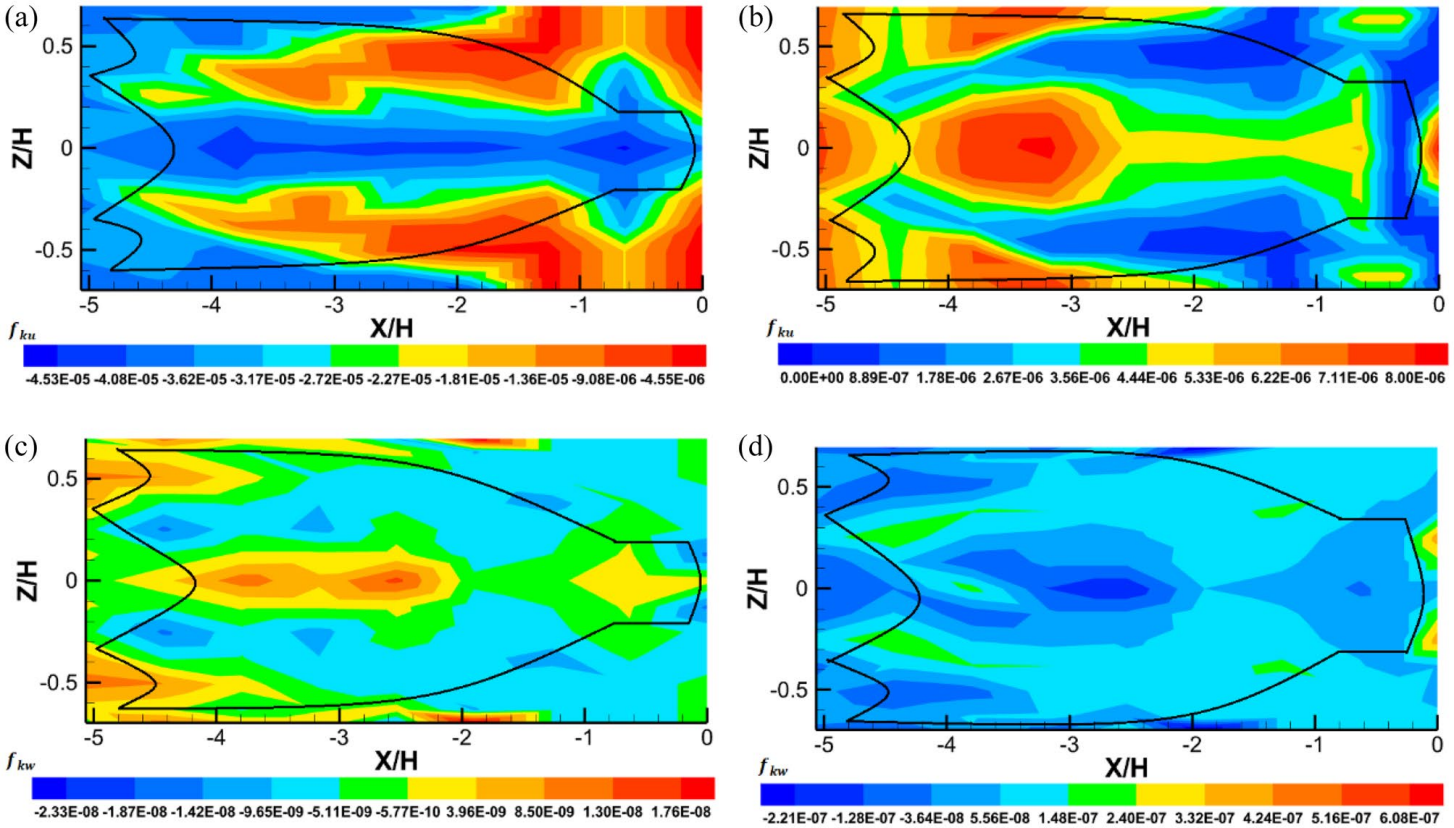
The gradient of turbulent kinetic energy flux components of the flow field inside and around midwater trawl net: Trawl without catch (**a** and **c**) and Trawl with catch (**b** and **d**).

Figure 10 compares the gradient of turbulent kinetic energy flux obtained inside and around the trawl net without catch and that obtained inside and around the trawl net with catch on the streamwise (${f}_{ku}$) and transverse (${f}_{kw}$) directions. Figure 10a shows that the maximum values of ${f}_{ku}$ (− 1.81×${10}^{-5}$ to − 4.56 × ${10}^{-6}$) observed inside and around the whole structure, indicating that the transport of turbulent kinetic energy is more important outside the trawl net and around the codend. The minimum values of ${f}_{ku}$ (− 4.53×${10}^{-5}$ to − 3.17 × ${10}^{-5}$) were distributed inside the trawl net along the centerline due to a higher flow velocities field (*u*) inside the trawl net (Fig. 10a). For the trawl with catch, a maximum distribution of ${f}_{ku}$ (4.44×${10}^{-6}$ to 8.01 × ${10}^{-6}$) was obtained inside the trawl body, around the trawl body near the structure (− 4.43 < *X/H* < − 2.53), and behind the end-part of the codend. However, inside and around the trawl body (near to the structure) and the codend (near the catches), the ${f}_{ku}$ was distributed uniformly with lower values (0 to 2.67 × ${10}^{-6}$) (Fig. 10b). In these figures, the maximum value of ${f}_{ku}$ indicates that the kinetic energy flux moves from upstream to downstream in the case of trawl with catch.

The gradient of turbulent kinetic energy flux in the vertical direction (${f}_{kw}$) was distributed with the negative values inside and around the trawl net without catch, indicating that the kinetic energy flux was in a downward direction. While the positive values of ${f}_{kw}$ were obtained inside and around the lower and upper side of the trawl wing (− 5.06 < *X/H* < − 4.43) and the trawl body (− 4.43 < *X/H* < − 3.16) indicating that the turbulent kinetic energy was moving in normal direction (Fig. 10c). For the trawl with catch, the ${f}_{kw}$ value were positives inside and around the whole trawl net. However, the negative values were observed inside the trawl wing and trawl body (− 5.06 < *X/H* < − 1.89), as well as inside and around the codend (− 1.12 < *X/H* < 0). These positive values and negative values of ${f}_{kw}$ mean that the gradient of turbulent kinetic energy was transported upstream in an upward direction due to instability in the flow inside and around this trawl.

#### Analysis of the turbulent kinetic energy

**Figure 11:**
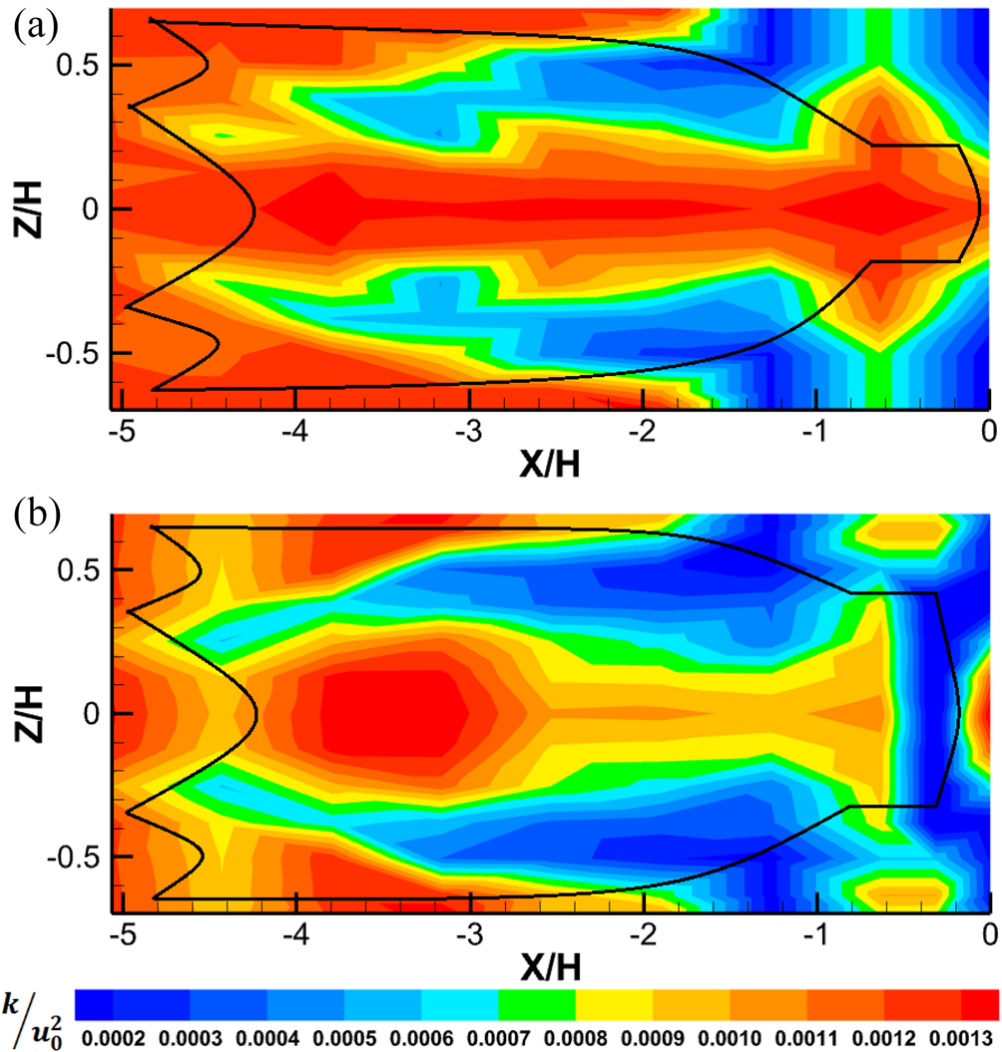
Turbulent kinetic energy ($k/u_{0}^{2}$) develops inside and around midwater trawl net (**a**) Trawl without catch and (**b**) Trawl with catch represented as left to right: front view and bottom view.

The turbulent kinetic energy (TKE) is a marker of the extension of unsteady turbulent perturbation inside and around the midwater trawl net and gives an evaluation of the unsteady turbulent content produced in the flow (Fig. 11). For the trawl without catch, the TKE was greater inside the trawl net mainly in the central plane of the trawl net, outside the trawl wing and the first seven sections of the trawl body in the wake zone (− 5.06 < *X/H* < − 1.89). These maximum values of TKE varied on average from 1.17×${10}^{-3}$ to 1.33×${10}^{-3}$ and were 73.01% greater than those obtained inside and around the other part of the trawl (Fig. 11a). For the trawl net with catch, TKE values vary from 5.42×${10}^{-4}$ to 1.13×${10}^{-3}$ (Fig. 11b). However, the greater values of TKE were inside the trawl body (− 3.86 < *X/H* < − 2.87), and behind the end-part of the codend on the central plane of the trawl net. In addition, due to the greater vertical pressure acting on the codend and the last part of the trawl body, and higher motions of these parts of the trawl net and the flow disturbance, lower TKE levels were observed in this part of the trawl net, and inside the codend with the values varying between 0 and 3.03×${10}^{-4}$ (Fig. 11b). On average, the trawl with catch has a greater TKE than the trawl without catch; and is 15.96% greater than those obtained inside and around trawl without the catch (Fig. 11).

### Analysis of the turbulent flow parameters inside and around the midwater trawl net

#### Spectral analysis of flow velocity field

**Figure 12:**
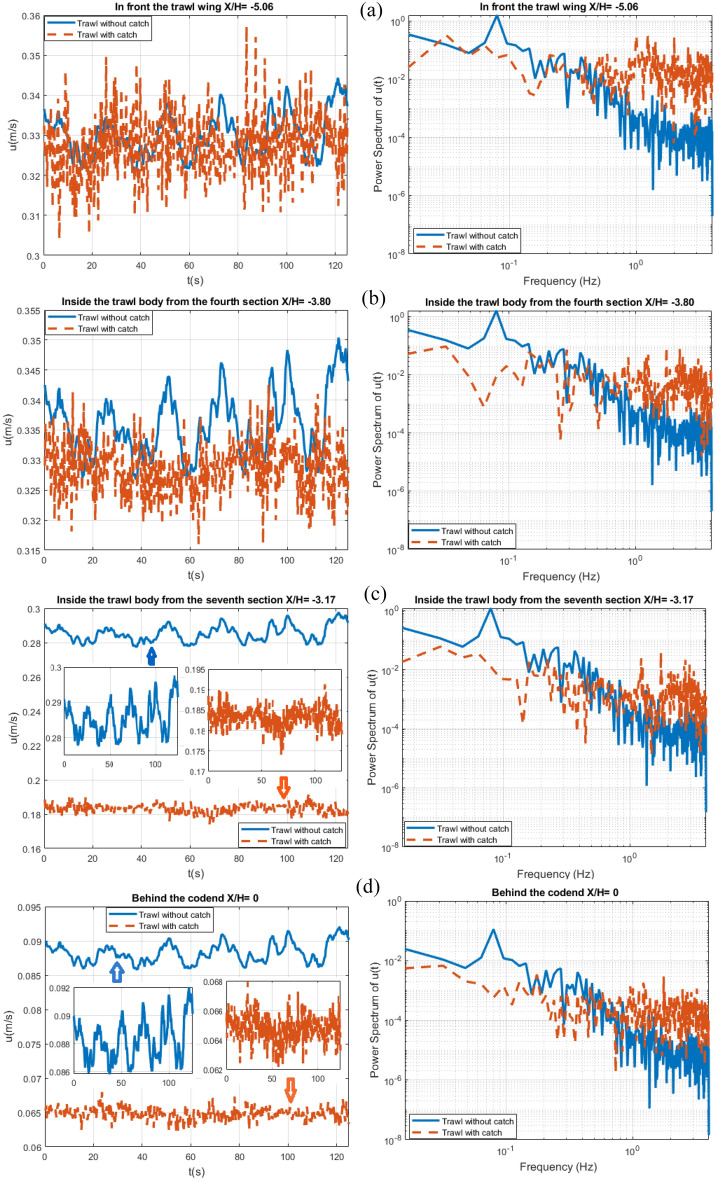
Time evolution of the streamwise velocity (right) linked to the normalized frequency spectrum (left in a log–log scale) at (*Y/H* = *0* and *Z/H* = *0.506*) represented as: (**a**) In front of the trawl wing (*X/H* = *− 5.06)*, (**b**) Inside the fourth section of trawl body (*X/H* = *− 3.80)*, (**c**) Inside the seventh section of trawl body (*X/H* = *− 3.17)*, and (**d**) Behind the codend (*X/H* = *0).*

**Figure 13:**
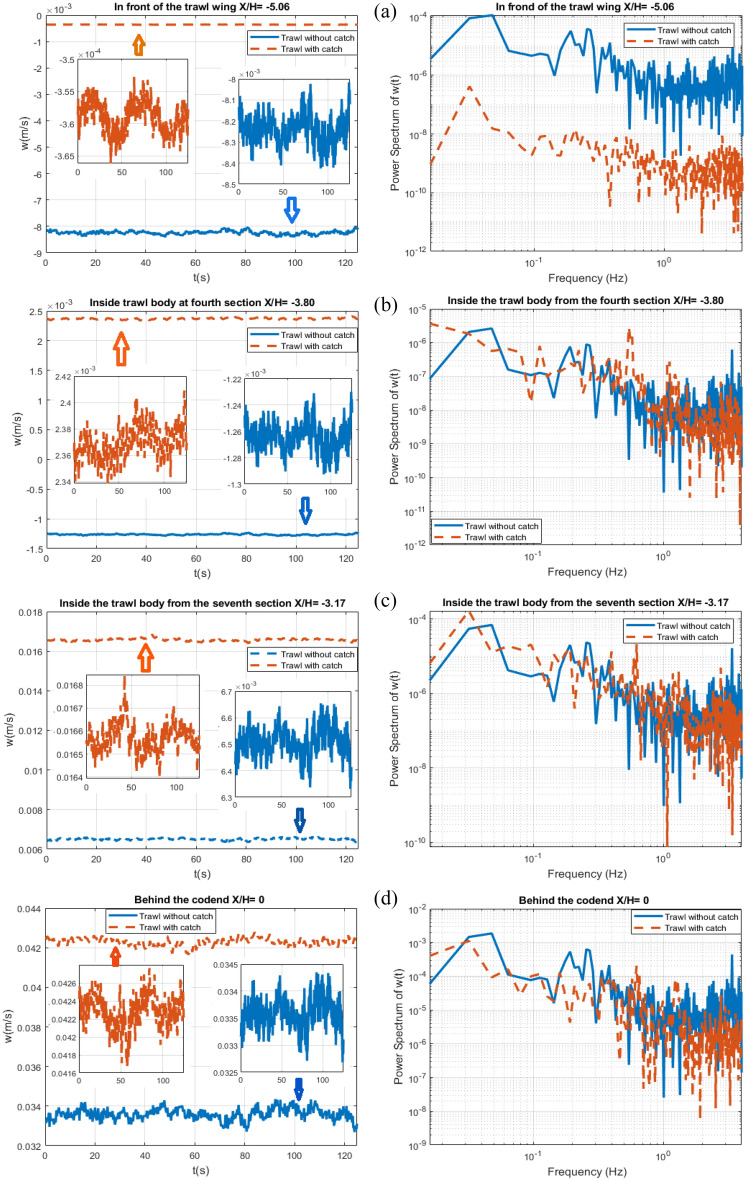
Time evolution of the transverse velocity (right) linked to the normalized frequency spectrum (left in a log–log scale) at (*Y/H* = *0* and *Z/H* = *0.506*) represented as: (**a**) In front of the trawl wing (*X/H* = *− 5.06)*, (**b**) Inside the fourth section of trawl body (*X/H* = *− 3.80)*, (**c**) Inside the seventh section of trawl body (*X/H* = *− 3.17)*, and (**d**) Behind the codend (*X/H* = *0).*

Figures 12 and 13 compares the time evolution of the flow velocity components (*u*, *w*) and the associated spectrum inferred from the ECVM database between the trawls without catch and with catch, in front of the trawl wing (*X/H* = − 5.06, *Y/H* = 0, and *Z/H* = 0.506), inside the trawl body from the fourth section(*X/H* = − 3.80, *Y/H* = 0, and *Z/H* = 0.506), inside trawl body from the seventh section (*X/H* = − 3.17, *Y/H* = 0, and *Z/H* = 0.506), and behind the codend (*X/H* = 0, *Y/H* = 0, and *Z/H* = 0.506). The temporal evolution of the flow velocities for both the trawls without catch and with catch present an oscillation of quasi-periodic nature, highlighting the unsteady nature of the flow field inside and around the different parts of the midwater trawl net. For the trawl net without catch, the maximum magnitude of the oscillations of the streamwise velocity is 3.8 × ${10}^{-3}$, 3.05×${10}^{-3}$, 2.7 × ${10}^{-3}$, and 1.17×${10}^{-3}$ m/s in front of the trawl wing, inside the trawl body from the fourth section, inside the trawl body from the seventh section, and behind the codend, respectively. The transverse velocity is 2.2 × ${10}^{-4}$, 3.35×${10}^{-5}$, 1.53 × ${10}^{-4}$, and 7.47×${10}^{-4}$ m/s in front of the trawl wing, inside the trawl body from the fourth section, inside the trawl body from the seventh section, and behind the codend, respectively (Figs. 12 and 13). For the trawl with catch, the magnitude of the oscillation of the streamwise velocity varies from 0.005 to 0.016 m/s, while for the longitudinal velocity, varies from 5.2×${10}^{-6}$ to 3.26×${10}^{-3}$ m/s. These magnitudes of the oscillations are greater for the temporal velocity measured behind the codend, than those obtained inside the trawl body and in front of the trawl wing (Figs. 12 and 13). For the trawl without catch, the values of the two component flow velocities inside the trawl body from the fourth section are 1.49%, 15.18%, and 73.81% greater than that obtained in front of the trawl wing, inside trawl body from the seventh section, and behind the codend, respectively. For the trawl with catch, they are 0.31%, 44.21%, and 88.18% greater than those in front of the trawl wing, inside the trawl body from the seventh section, and behind the codend, respectively (Table 2).

**Table 2:** Mean flow velocities of the two components.

Part of trawl net	Trawl without catch		Trawl with catch	
	*u*	*w*	*U*	*w*
Trawl wing	0.331 $\pm$ 0.032	− 0.0082 ± 0. 0002	0.327 $\pm$ 0.089	− 0.0004 $\pm$ 0.00004
Trawl body from the fourth section	0.336 $\pm$ 0.029	− 0.0013 ± 0.0004	0.328 $\pm$ 0.094	0.0024 ± 0.0043
Trawl body from the seventh section	0.285 ± 0.088	0.0065 ± 0.004	0.183 $\pm$ 0.009	0.017 ± 0.002
Behind the codend	0.088 ± 0.0048	0.0335 ± 0.0058	0.065 ± 0.0089	0.043 ± 0.0017

For the trawl without catch, the highest frequency peak representing the periodic flow is obtained at a very lower frequency ($f^{\prime}_{1}$) 0.08 Hz and 0.37 Hz for the streamwise and transverse velocities, respectively on all the target points. For the trawl with catch, the FFT results of the variation of the streamwise flow velocity showed that the highest frequency peak was attained at very low frequency components of $f^{\prime}_{1}$= 0.032 Hz on all the target points. However, for the transverse velocities, the highest frequency peak was attained at $f^{\prime}_{1}$= 0.032 Hz for all the target points except inside the trawl body from the fourth section, which was obtained at $f^{\prime}_{1}$= 0.016 Hz (Figs. 12 and 13). The second frequency peak is observed at $f^{\prime}_{2}$= 0.16 Hz for both streamwise and transverse velocities on all the target points for the trawl without catch. For the trawl with catch, ${f^{{\prime}}}_{2}$= 1.008, 0.88, 1.23, and 0.46 Hz in front of the trawl wing, inside the trawl body from the fourth section, inside the trawl body from the seventh section, and behind the codend, respectively.

When regarding the raw spectra, the amplitude of the streamwise velocity spectra is 1000 and 100 times greater than that of transverse velocity spectra for the trawl without catch and with catch, respectively. Furthermore, the power spectrum content of both the streamwise and transverse velocities obtained on the trawl with catch is about 80.37% lower than that obtained on the trawl without catch. The result of the power spectrum content obtained with the streamwise velocities presents quasi-periodic oscillations corresponding to a global mean flow. The transverse velocities also present quasi-periodic oscillations and can be linked to the time evolutions of velocities imposed by the unsteady turbulent motions on midwater trawl net.

#### Spectral analysis of Turbulent kinetic energy and Reynold shear stress

**Figure 14:**
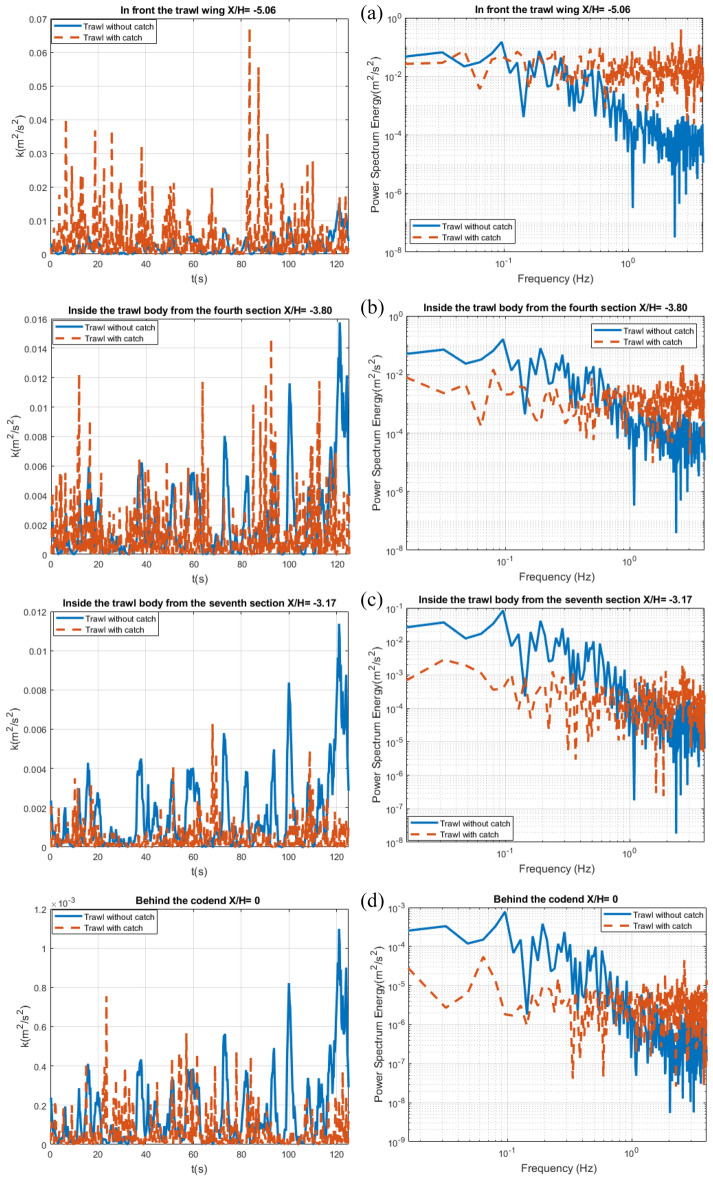
Time evolution of the TKE (right) linked to the normalized frequency spectrum Energy (left in a log–log scale) at (*Y/H* = *0* and *Z/H* = *0.506*) represented as: (**a**) In front of the trawl wing (*X/H* = *− 5.06)*, (**b**) Inside the fourth section of trawl body (*X/H* = *− 3.80)*, (**c**) Inside the seventh section of trawl body (*X/H* = *− 3.17)*, and (**d**) Behind the codend (*X/H* = *0).*

The temporal signals of the time evolution of the TKE presents temporal oscillations corresponding to the passages of the unsteady turbulent flow through the midwater trawl net for both the trawl without catch and the trawl with catch (Fig. 14). The presence of the catch inside the trawl lead to an increase in oscillation amplitude of about 94.21%, 72.85%, 41.31%, and 35.22% for the TKE oscillations obtained in front of the trawl wing, inside the trawl body from the fourth section, inside trawl body from the seventh section, and behind the codend, respectively, compared to those obtained on the trawl without catch (Fig. 14).

Each spectrum associated with the temporal signal presents two main peaks. The first, being: ${f^{{\prime\prime}}}_{1}$= 0.096 Hz and the second, being a maximal frequency peak corresponding to ${f^{{\prime\prime}}}_{2}$= 0.57 Hz for all the temporal signals of the TKE oscillations inside and outside the different parts of the trawl without catch (Fig. 14). For the trawl catch, the frequency of the TKE inside and around its different parts indicated several peaks when using the FFT method as shown in Fig. 14. The spectrum exhibits a first maximal frequency peak corresponding to low frequencies of ${f^{{\prime\prime}}}_{1}$ =0.048, 0.08, 0.032, and 0.064 Hz in front of the trawl wing, inside the trawl body from the fourth section, inside the trawl body from the seventh section, and behind the codend, respectively. The second peak was obtained at ${f^{{\prime\prime}}}_{2}$= 2.67 Hz for all the temporal signals of the TKE oscillations. These main peaks are associated with periodic oscillations of releases of unsteady turbulence in the wake of the two trawls. This phenomenon is called "lock-in".

**Figure 15:**
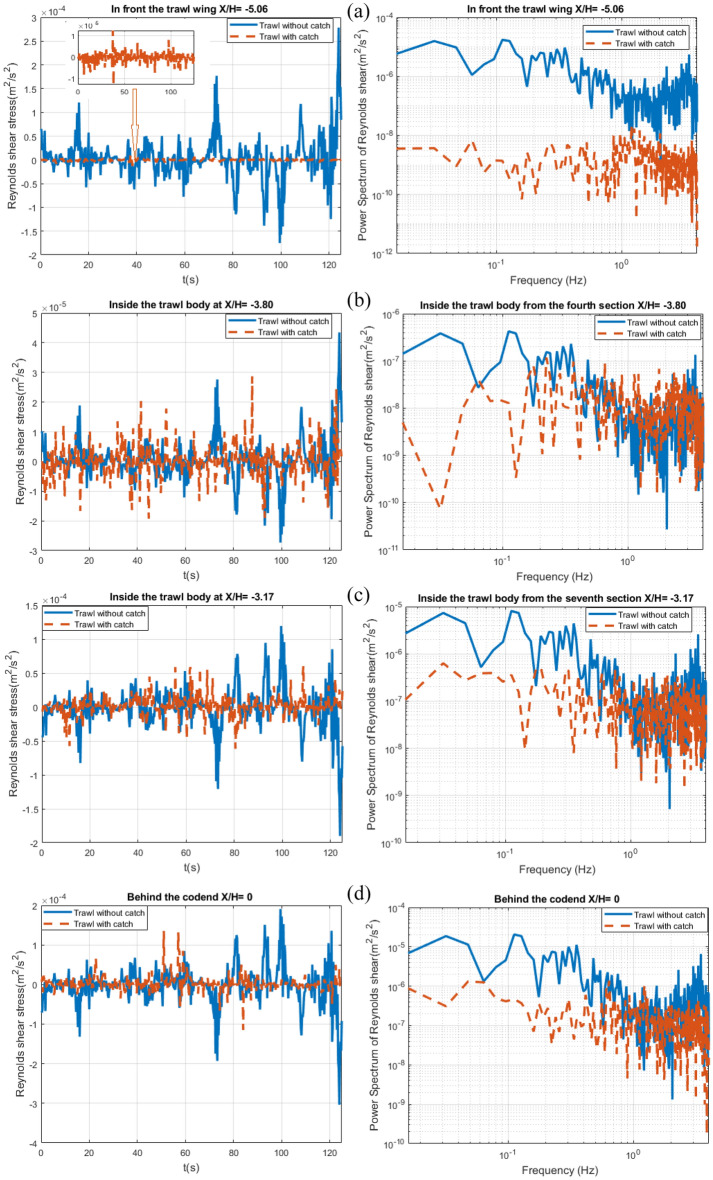
Time evolution of the Reynolds stress shear component $\overline{u^{\prime}w^{\prime}}$ (right) linked to the normalized frequency spectrum (left in a log–log scale) at (*Y/H* = *0* and *Z/H* = *0.506*) represented as: (**a**) In front of the trawl wing (*X/H* = *− 5.06)*, (**b**) Inside the fourth section of trawl body (*X/H* = *− 3.80)*, (**c**) Inside the seventh section of trawl body (*X/H* = *− 3.17)*, and (**d**) Behind the codend (*X/H* = *0).*

The temporal evolution of the momentum flux (Reynolds stress shear) shows quasi-periodic oscillation with significant amplitude variations (Fig. 15). The maximum amplitude values of these oscillations range from 4.2×${10}^{-5}$ to 2.6×${10}^{-4}$ ${\mathrm{m}}^{2}/{\mathrm{s}}^{2}$ and 1.3×${10}^{-5}$ to 1.46×${10}^{-4}$ ${\mathrm{m}}^{2}/{\mathrm{s}}^{2}$ inside and around the different parts of the trawl without catch and with catch, respectively. The oscillation amplitudes of the temporal evolution of the momentum flux behind the codend are higher than those obtained inside or outside other parts of the trawl net ; they are 15.30%, 84.61%, and therefore 46.15% greater than those obtained in front of the trawl wing, inside the trawl body from the fourth section, and inside the trawl body from the seventh section, respectively, for both the trawl without catch and with catch.

For the trawl without catch, the highest frequency peaks were obtained at two different frequencies representing the periodic flow. These peaks were obtained at a very lower frequencies of ${f^{{\prime\prime\prime}}}_{1}$= 0.032 Hz and ${f^{{\prime\prime\prime}}}_{2}$= 0.35 Hz on all the target points. For the trawl with catch, the highest frequencies peaks were obtained at frequencies of ${f^{{\prime\prime\prime}}}_{1}$= 0.064, 0.041, 0.032, and 0.048 Hz in front of the trawl wing, inside the trawl body from the fourth section, inside the trawl body from the seventh section, and behind the codend, respectively. The second peak (${f^{{\prime\prime\prime}}}_{2}$) was obtained at 1.2, 1.08, 0.75, and 0.67 Hz in front of the trawl wing, inside the trawl body from the fourth section, inside the trawl body from the seventh section, and behind the codend, respectively (Fig. 15). The trawl with catch exhibited a waving motions and lower fluctuating velocity part and power spectrum on Reynolds stress shear variation compared to the trawl without catch.

#### Variation of turbulent length scale and the turbulent Reynolds number in unsteady turbulent flow inside and around midwater trawl

**Figure 16:**
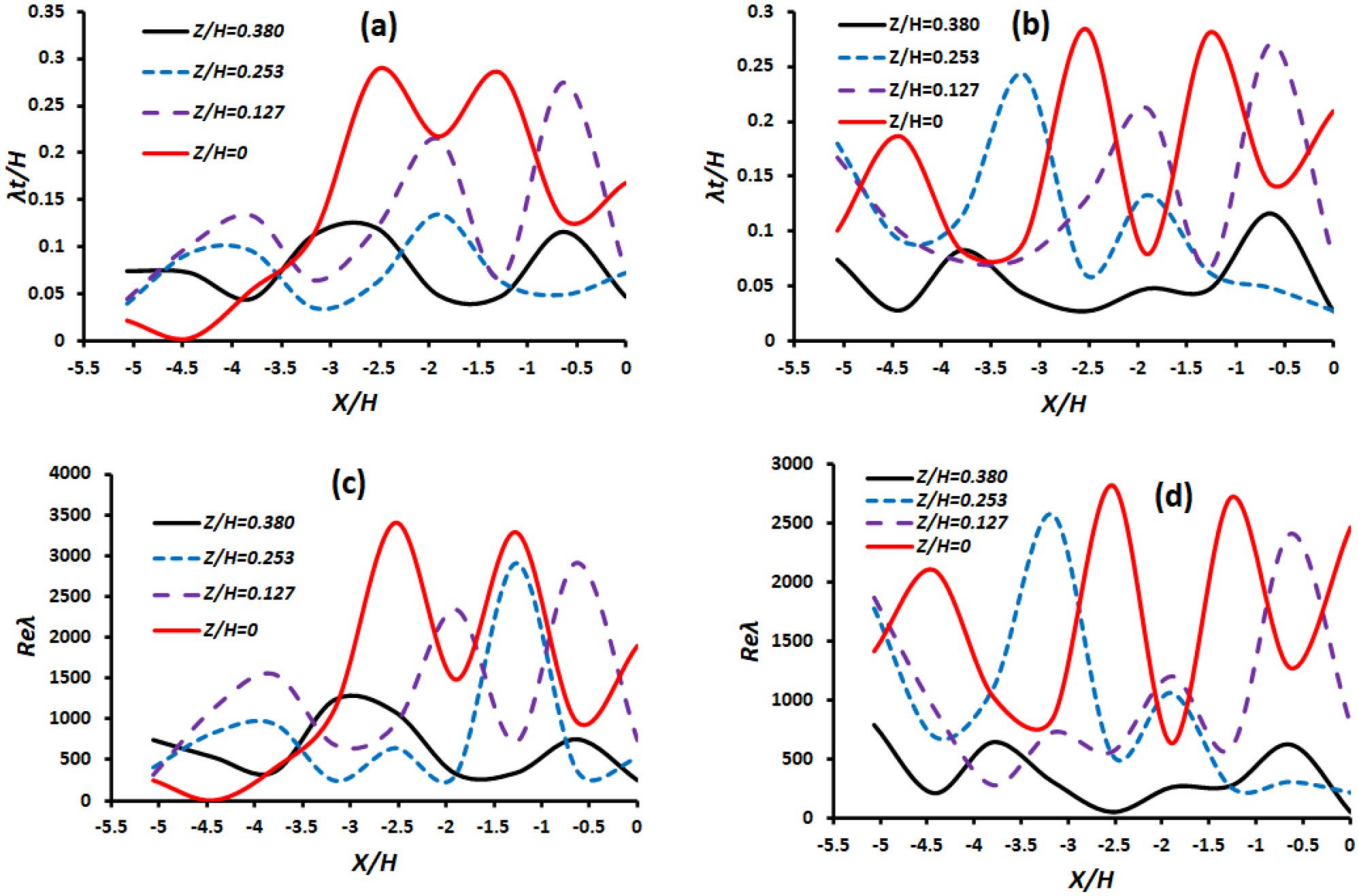
Plots of the normalized Taylor microscale (Upstairs) and Taylor-scale Reynolds number (Downstairs) against *X/H* at different *Z/H* and* Y/H* = 0: (**a** and **c**) Trawl net without catch, and (**b** and **d**) Trawl net with catch.

Figure 16a,b show that the normalized turbulent length scale ${\uplambda }_{\mathrm{T}}$ increase as *Z/H* increases and is associated with the reduction of ${\sigma }_{u}$. At the centerline(*Z/H* = 0) of the trawl without catch, ${\uplambda }_{\mathrm{T}}$ increase as *X/H* increases from − 5.06 (in front of the trawl wing) to − 2.53 (inside the trawl body from the seventh section) and attend peaks at *X/H* =− 2.53 and − 1.26. In this case, the unsteady turbulent is more important inside the trawl body from the seventh section, inside the codend, and behind the codend than other trawl parts. That is why the taylor-scale Reynolds number obtained inside the trawl body from the seventh section and the codend (− 2.53 < *X/H* < − 1.26) was about 92.68, 88.39, and 44.30% greater than that obtained in front of the trawl wing (*X/H* = − 5.06), inside the trawl body from the fourth section (*X/H* = − 3.79), and behind the codend(*X/H* = 0), respectively (Fig. 16c). For the trawl with catch, ${\uplambda }_{\mathrm{T}}$ and ${R}_{\uplambda }$ reach the maximum value at *X/H* = − 2.53(inside the trawl body), -1.26 (inside the codend), and 0 (behind the codend) at *Z/H* = 0 and 0.127. Therefore, at *Z/H* > 0.127, the unsteady turbulent of the flow passage was lower at *X/H* > − 2.5 compared to other parts of the trawl. These was because ${\uplambda }_{\mathrm{T}}$ and ${R}_{\uplambda }$ were lower at *X/H* > − 2.5 (inside the trawl body from the seventh section and inside and behind the codend) unlike at X/H < − 2.5 (inside and around the trawl wing and trawl body from the fourth section (Fig. 16b,d). In addition, the mean ${\uplambda }_{\mathrm{T}}$ and ${R}_{\uplambda }$ obtained in the different parts of the trawl net with catch were about 15.98%, 51.11%, 34.12%, and 30.81% in front of the trawl wing, inside the trawl body from the fourth section, inside the trawl body from the seventh section, and behind the codend, respectively, greater than those obtained on the trawl net without catch.

## Discussion

This study analyzed the impact of the catch on the behavior and the characteristics of the turbulent flow inside and around the midwater trawl using 3D ECVM measurements obtained at several points inside and outside the trawl net structure. This analysis showed that the turbulent intensity inside and around the midwater trawl varied from 0.31 to 4.01% and 0.66 $\mathrm{to}$ 4.88% for the trawls without catch and with catch, respectively. These turbulence intensities were ranged according to the interval of those computed in the same flume tank, but without trawl net by Thierry et al.^23^ and Hu et al.^36^. The above confirmed the turbulent nature of the flow in the flume tank. Similar observations were found on the studies of Bouhoubeiny et al.^12^, and Druault and Germain^13^ during flow measurement around the moving trawl net in the flume tank at IFREMER (French Research Institute for Exploitation of the Sea).

However, the Reynolds number as a function of twine diameter was 4071.6 confirming the development of the turbulent flow inside and around the midwater trawl. Thus, according to the studies of Druault et al.^24^, Bouhoubeiny et al.^25^, and Kim^30^, this turbulent flow is composed by the turbulent boundary layer flow that develops inside and around the trawl body near the upper and the lower side of the structure, and also, due to the turbulent flow on the trawl wake which develops inside the trawl net. Furthermore, using the codend diameter (0.11 m and 0.32 m for the trawls without catch and with catch, respectively) the Reynolds number was 59,208.3 and 172,196 for the trawls without catch and with catch, respectively^11,13^. This Reynolds number confirms the presence of the vortex shedding around the codend created by the unsteady motions of the trawl net and is more important for the trawl with catch. According to the research of O’Neill et al.^11^ regarding the analysis of the effect of catch size on the codend drag, and that of Druault and Germain^13^ on the analysis of hydrodynamics of the moving trawl codend and its fluttering motions, the presence of the catch inside the trawl net results in a larger codend volume and blockage of the meshes, which limits the flow passage through the trawl structure and generates a greater transverse drag pressure on the trawl net. This transversal drag pressure produced the unsteady motions on the three axes causes a strong reduction in the flow velocities, which in turn cause a flow disturbance, creating the vortex shedding inside the trawl near the catches and around the trawl codend^6,12,13,47^.

The development of this unsteady turbulent flow inside and around the midwater trawl net, strongly depends on the catch and the nature of the trawl motions. Indeed, the velocity reduction inside and around the trawl without catch and with catch varies from 1.41 to 74.15% and 0.72 to 85.43% and the closer the proximity to the codend, the more this velocity reduction increases. The above reason being, the closer we get to the codend, the smaller the mesh size and the tilt angle becomes. This disrupts the flow passage through the trawl mesh and causes the development of an unsteady turbulent flow. The development of the unsteady turbulent flow can be also explained by the fact that the presence of the catch inside the trawl net increased the transverse and longitudinal motions, and considerably modified the mesh opening, resulting in a decrease in the flow passage^6,24,25^. The notion that the velocity reduction increased with decreasing tilt angle and mesh opening was demonstrated in the flow study around the fishing net by Bouhoubeiny et al.^25^, Druault et al.^20^, and Bi et al.^48^. This trend was also confirmed by the study of the turbulent flow around the bottom trawl net by Druault et al.^24^, and Thierry et al.^14^.

Furthermore, the greater velocity deficit inside and around the trawl net with the catch can be explained by the fact that the presence of the catch in the trawl net blocks the mesh opening and deforms the trawls shape^11,49,50^. This trawl deformation and the mesh opening blockage disrupts the free passage of the flow due to the occurrence of energy exchange phenomena, due to the turbulent kinetic energy. This energy exchange phenomena is because of the creation of the TKE although it is more important inside and outside the trawl with catch, it also exist through the trawl without catch due to its motions and the nature of its structure. These energy exchange phenomena give rise to the creation of unsteady turbulent flow (turbulent boundary layer flow and vortex shedding) in the trawl wake, which considerably increases the water drag pressure on the trawl. This increase in the water drag pressure due to the catch and trawl motions affects the overall trawl drag and varies as a function of the trawl position^50,51^.

Regarding the trawl motions, this study has demonstrated that the trawl with catch has a greater instability with large amplitude transverse oscillations compared to the motions of the trawl without catch. This trend was confirmed by Liu et al.^49^ demonstrating that midwater trawl codend without catch had lower transverse oscillations. The main reason could be the presence of the catch that reduces the water filtration through the trawl codend leads to the formation of vortex flow inside the codend, and intense trawl oscillations. However, for the streamwise oscillation, the trawl with catch also showed a more unsteady behavior with an amplitude 50% greater than that of the trawl without catch. This unstable behaviour of the trawl motions (transverse and streamwise motions) on the trawl with catch, can be explained by the fact that the presence of the catch inside the trawl net can lead to a larger volume covered by the trawl net, which will allow the unsteady turbulent flows to develop inside and around this structure and generate a great drag pressure on the trawl^23,52^. This pressure is more important on the trawl with catch than on the trawl without catch as it influences the amplitude of the trawl oscillation due to the frequency of the turbulent flow. Thus, the fact that the trawl had catches inside, allowed it to increase its unsteady oscillatory motions and lead to a reduction in the trawl selectivity. This is due to a decrease in the fish swimming performance inside the trawl because of the presence of the vortex shedding, which limits the flow diffusion through the trawl^11,13,50^. That is why, according to Kim^10,30^ and Druault et al.^20^, the knowledge of the transverse and streamwise motions of the trawl net is very essential for understanding the fluid–structure interaction of this fishing gear in terms of the improvement of profitability and ensuring the sustainability of the resources, and the by-catch selection.

The present study showed that the detection signal of the transverse and streamwise motions of the two trawl nets exhibited low frequencies. These frequencies, and ${f}_{1}$ in particular, were lower than those linked to the fishing net oscillations by Bouhoubeiny et al.^25^ and Druault et al.^20^ using TRPIV. They were also lower than the frequencies associated with the bottom trawl net motions obtained by Druault et al.^24^ using particle image velocimetry (PIV). The difference between these studies is simply due to the low frequency on the trawl net being independent of the frequency of the streamwise flow velocity^13^. When the dimensionless Strouhal number ${S}_{t}$ = $\frac{fd}{{u}_{0}}$ is calculated based on these frequency values, one obtains ${S}_{t1}$ = $\frac{{f}_{1 }d}{{u}_{0}}$  = 0.0024 and 0.0028 and ${S}_{t2}$ = $\frac{{f}_{2 }d}{{u}_{0}}$  = 0.11 and 0.14 for the trawl without catch and with catch, respectively. Comparing these Strouhal numbers with those obtained by Druault and Germain^13^, the ${S}_{t1}$ for both the trawls without catch and with catch was lower than those obtained by them using the lower frequency, but ${S}_{t2}$ for the trawl with catch were greater than that obtained by using the second frequency peak. The fact that the low frequencies of the trawl with catch were lower than those obtained on the trawl without catch demonstrated that the unsteady turbulent flow frequencies of the trawl with catch are lower than those of the trawl without catch. Therefore, the presence of both liner and catch greatly influences the unsteady flow behaviors. This hypothesis confirms that suggested by Druault and Germain^13^ and Bouhoubeiny et al.^12^ on the codend motions demonstrating that for flow around the codend, the vortex shedding frequency of a moving structure is lower than that of a structure at rest. Conversely, the first peak of the trawl motions can be due to higher structural vibrations on the trawl with catch, and the second peak could be due to the periodic oscillations induced by turbulent boundary flow and vortex shedding inside and around the two trawl structures^13^. By observing these results and those obtained by Facq et al.^53^ and Bouhoubeiny^51^, it is clear that low-frequency motion seems to be directly linked to the cyclical variations in the trawl drag and associated with the elastic nature of its structure. Finally, in the flume tank, both the trawl without catches and with catch will vibrate because of the development of unsteady turbulent flow and the bridle tension that maintains the trawl net horizontally. Thus, the trawl oscillation motions depend on the difference between the frequencies associated with both phenomena leading to the creation of the TKE, and the oscillation caused by bridle tension that at most dominant depending on the catches. Therefore, the results of this study highlight the coupling between the unsteady turbulent flow sheet and the low frequencies detected in the fluttering motion of the midwater trawl.

Regarding the Reynolds stress tensor, the normal turbulence stresses (${\overline{u^{{\prime}}}^{2}}$) were greater inside the midwater trawl net on the central section, near the first-part of the trawl body, and in front of the trawl wing. Unlike, ${\overline{w^{{\prime}}}^{2}}$ the shear stresses ($\overline{u^{{\prime}}v^{{\prime}}}$) have high values around the trawl body from the seventh section near the structure and behind the codend for the trawl without catch. On the other hand, they have higher values inside and around the trawl with catch when compared to those of the trawl without catch, particularly, around the trawl body and behind the codend. These higher values around the trawl with catch are due to the drag pressure exerted by the vortex shedding on the free surface of the catch and the unsteady turbulent boundary layer flow caused by weak flow passage through the trawl net. The above is in addition to the presence of the trawl liner and the angle between the flow direction and trawl net, as reported by Bouhoubeiny et al.^25^ and Druault and Germain^13^. This indicates that a large proportion of the turbulent stress is created by the formation of the TKE, turbulent flux, and the gradient of turbulent kinetic energy flux through the trawl with catch. This formation of turbulent parameters is of great energetic importance compared to other fluctuations. Conversely, the value obtained in the present study is lower than that obtained by Bouhoubeiny et al.^12^ around the rigid codend and Druault and Germain^13^ in the downstream region of the fluttering codend. The reason for this is that the present study is based on the flow inside and around the whole trawl net, unlike that conducted by Bouhoubeiny et al.^12^ and Druault and Germain^13^. Another reason for this is that the difference in the trawl net type, streamwise velocity, experiment process, and Reynolds number means a higher Reynolds number leads to an increase in the flow velocity fluctuation and a higher Reynolds stress^23,54^.

Spectral analysis of *u* and *w* in front of trawl wing, inside trawl body, and behind the codend of both the trawl without catch and with catch showed that higher frequency peaks were obtained at the very lower frequency components. These frequency components are close to the frequencies linked to the trawl motions (${f}_{1}$ and ${f}_{2}$) and those linked to Reynolds stress and TKE in the shear layer of the unsteady turbulent flow. Such a shear layer covers the entire surface swept by the trawl structure and it is more important for the codend because of its intense motion. Note, that this shear layer will also depend on the catch, and thus more prevalant on the trawl with catch, compared to that of the trawl without catch. Thus, in this shear layer, particularly, on the trawl with catch, the subharmonics of the main low frequency (${f}_{1}$=$f^{\prime}_{1}$. = s$f^{\prime\prime}_{1}$. =$f^{\prime\prime\prime}_{1}$) of the trawl oscillation were observed. This indicated that the main turbulent flow instabilities inside and around the trawl on the shear layer originate from trawl motions and the presence of catches inside the trawl net. This observation was similar to those obtained using the spectral analysis of *u* on the codend and the fishing net by Druault and Germain^13^ and Druault et al.^20^, respectively. The results of this study found that the transverse velocity component of the trawl with catch was greater than that of the trawl witht catch, reason being the velocity component in each part of the two trawls was related to the unsteady turbulent flow frequency ($f_{2}$ close to $f^{\prime}_{2}$), which was more influenced by the presence of the catches. The results confirm the hypothesis in the turbulent boundary layer flow and the vortex flow frequencies coincide with the structure's oscillations. This was also confirmed by the results of the power spectrum energy and the power spectrum of the Reynold shear stress. In comparison to the results of the power spectrum content, the low-frequency signal on the vortex shedding behind the codend by Bouhoubeiny et al.^12^ and Druault and Germain^13^, was consistent with signals obtained in this study than inside the trawl body from the seventh section and behind codend, and was linked to the strong trawl oscillations. In addition, the second frequency peak ($f^{\prime}_{2}$) obtained in this study was consistent with those obtained by Druault et al.^24^ around the bottom trawl, Bouhoubeiny et al.^25^, and Druault et al.^20^ around the horizontal part of the fishing net on the detection of the unsteady turbulent boundary layer flow around trawl net and fishing net structure. This frequency peak value is lower than previous works dealing with a wall-mounted cylinder wake vortex-shedding frequency by Ikhennicheu et al.^55^. This is because the flow around the wall-mounted cylinder is influenced only by its rigid structure whereas that of the trawl net depends on the structure vibration caused by the catch, its motions, and its flexibility.

## Conclusion

3D ECVM measurements were carried out to examine the turbulent structure and to characterize the flow organization inside and around 1/35 scale midwater trawl models. The goal of this work was to analyze the impact of catch on the development of the unsteady turbulent flow and to attain a better understanding of the fluid–structure interaction of the midwater trawl fisheries. The Reynolds stress tensor, turbulent flux, the gradient of turbulent kinetic energy, and TKE were determined and analyzed from the ECVM databases. Fourier analysis, statistical and instantaneous unsteady turbulent flow identification analysis were successively used to investigate the flow velocity field, the TKE, momentum flux, and trawl motions. The following principal conclusions can be drawn:

1. The analysis of time-averaged flow velocities clearly shows that the mean flow characteristics depends greatly on the trawl motions and the presence of the catches inside the gear. Indeed, the velocity deficits inside and around the trawl with catch was greater compared to that obtained inside and around the trawl without catch. This because the vertical motion oscillations of the trawl with catch were intensely shaken and higher compared to that of the trawl without catch.
2. We assumed that the fluttering oscillatory motions of the trawl net are in the transverse and streamwise directions, and were influenced by the catch. The motion of the trawl net exhibited dominant low-frequency components superimposed by another component associated with the unsteady turbulent flow street. These trawl motions modified the drag force acting on the trawl and the local porosity of the codend. Thus, because the flow velocity measurements were only made for a specific flow pattern with a 1/35 scaled trawl model, it is expected that in full-scale the amplitude of the trawl oscillations can be 35 times higher during fishing operations and the transverse motion of the order of one thirty fifth the codend diameter with catch inside.
3. The periodic unsteady turbulent flow is significantly influenced the transverse and streamwise motions of the trawl net, which will affect the fluid flow inside and around the trawl net, fluid force, and turbulent flow model. Therefore, a complex interaction between the fluid, trawl structure, and catches is present. The present study showed that the flow seems to recover a two-dimensional state, and large-scale energetic flow structures at are identified very low frequency.
4. Analysis of TKE and higher-order moments have highlighted that the TKE is mainly evident inside and around the trawl wing and the first part of the trawl body as compared to other part of the trawl net, and the gradient of turbulent kinetic energy flux is greater on the trawl codend. In addition, TKE exchanges was lower inside the trawl with catch, in comparison with the trawl net without catch. Thus, the distribution of the turbulent fluctuation inside and around the trawl with catch was more important.
5. The present study is an experiment aimed at reproducing the entire trawl hydrodynamics and linked to the trawl fluttering motions. The study makes it possible to observe the main flow characteristics developing inside and around an oscillating trawl. It is therefore expected that the current results, and in particular, the trawl motions will provide significant information that could be extrapolated to a full-scale trawl net under certain conditions.

## Data Availability

Data will be available upon request by the corresponding author Dr Hao Tang.
